# Effects of a 14-day social media abstinence on mental health and well-being: results from an experimental study

**DOI:** 10.1186/s40359-024-01611-1

**Published:** 2024-03-13

**Authors:** Lea C. de Hesselle, Christian Montag

**Affiliations:** Department of Molecular Psychology, Institute of Psychology and Education, Ulm University, Helmholtzstraße 8/1, 89081 Ulm, Germany

**Keywords:** Social media, Abstinence, Body image, FoMO, PSU, Loneliness

## Abstract

**Background and aim:**

The study investigated the effects of a 14-day social media abstinence on various mental health factors using an experimental design with follow-up assessment. Hypotheses included positive associations between problematic smartphone use (PSU) and depression, anxiety, fear of missing out (FoMO), and screentime. Decreases in screentime, PSU, depression and anxiety, and increases in body image were assumed for the abstinence group. Additionally, daily changes in FoMO and loneliness were explored.

**Methods:**

Participants completed different questionnaires assessing PSU, FoMO, depression and anxiety, loneliness and body image and were randomized into control and social media abstinence groups. Daily questionnaires over 14 days assessed FoMO, loneliness, screentime, and depression and anxiety. 14 days after the abstinence, a follow-up questionnaire was administered. Multilevel models were used to assess changes over time.

**Results:**

PSU was positively associated with symptoms of depression, anxiety and FoMO, but not with screentime. Spline models identified decreased screentime and body image dissatisfaction for the intervention group. Depression and anxiety symptoms, PSU, trait and state FoMO, and loneliness, showed a decrease during the overall intervention time but no difference between the investigated groups could be observed (hence this was an overall trend). For appearance evaluation and body area satisfaction, an increase in both groups was seen. Daily changes in both loneliness and FoMO were best modelled using cubic trends, but no group differences were significant.

**Discussion:**

Results provide insights into effects of not using social media for 14 days and show that screentime and body image dissatisfaction decrease. The study also suggests areas for future studies to better understand how and why interventions show better results for some individuals.

**Supplementary Information:**

The online version contains supplementary material available at 10.1186/s40359-024-01611-1.

## Background

Social media is part of everyday life with 4.76 billion users worldwide and a 3.0% annual increase [1]. The average time spent on social media is 2.5 hours, totalling 5 hours of screentime per day [1]. Simultaneously, there is a global rise in mental health issues with a 25% increase in anxiety disorders and a 28% increase in depressive symptoms, primarily affecting young adults [2]. It has already been discussed if the increase in social media use paved the way for the increase is psychopathologies, but establishing causality remains difficult [3]. Despite this, studies have linked problematic social media use to problems such as symptoms of depression and anxiety, [4–6] stress, negative body image and low physical activity [7–10].

While most studies use cross-sectional data, assessing changes over time in longitudinal data is necessary. The present study combines a longitudinal design and experimental approach to evaluate effects of a 14-day social media abstinence on several mental health factors.

### Problematic smartphone use (PSU)

Smartphones enable various activities (e.g., communication, entertainment, gaming, online surfing or using social media). Excessive smartphone use which can lead to adverse consequences has been termed problematic smartphone use (PSU, [11, 12]) This includes relying on the smartphone to regulate one’s mood, experiencing agitation in its absence, and unsuccessful attempts to reduce usage [11, 12].

PSU has been associated with different negative life and health outcomes such as poor sleep quality, [13–15] impaired work and academic performance, [16–19] neck and shoulder pain, [20, 21] and visual impairment [22, 23]. Further, PSU has been positively associated with depression, anxiety, and Fear of Missing Out (FoMO) [24–30]. Though simple cross-sectional associations do not allow causal interpretation, according to the Compensatory Internet Use Theory (CIUT, [31]) excessive smartphone use can be interpreted as a coping mechanism for dealing with life stressors and negative emotions. Seen this way: associations between negative affect and overuse of technology might exist due to “self-medication” principles, although such medical language needs to be further investigated regarding its fit in the realm of Internet Use Disorders [32]. Another theoretical framework which is often used in PSU research is the Interaction of Person-Affect-Cognition-Execution model (I-PACE, [33, 34]). This model describes different core characteristics and dispositional factors (personality, history of psychopathology, genetics, etc.) which can impact on how situations are received and what the response is (cognitive biases, certain affective responses), thereby contributing to the development of PSU. It offers insights into, for example, how stressful situations might lead to heightened smartphone use (in detail: use of certain applications) as a coping mechanism for dealing with stress. Of note, the I-PACE model also presents a history of psychopathologies such as depression as a vulnerability factor within the P-variable to develop excessive online use patterns. Hence, much of the variables investigated and introduced later in this manuscript could be seen through the lens of the I-PACE model: in particular, we mention that the intervention aiming at reduction of social media use could trigger changes in cognitive and affective processes which in turn might result in lower psychopathological tendencies as recorded via several variables in the present work (e.g. depressive tendencies or body image dissatisfaction).

Though most aforementioned studies assess PSU via self-report questionnaires, some others have used objective measurements of smartphone use (screentime, screen unlocks) and found either no association between depression and screentime [27] or an inverse relationship between depression and number of screen unlocks, [27, 35] indicating even a lower unlock frequency for depressed compared to non-depressed individuals. However, objective and subjective measures of smartphone use are only moderately associated [27, 36].

### Social media use

Social media use presents one specific form of spending (excessive) time on the smartphone as platforms enable users to share real-time pictures, videos, and other content, facilitating connections through likes, comments, and multimedia messages. A lot of daily smartphone screentime is spent on social media, potentially leading to adverse consequences. Problematic social media use (PSMU) shows itself in symptoms similar to PSU, however it applies especially to social media use. This includes maladaptive behaviours such as escalating time spent on social media and unsuccessful efforts to reduce usage, resulting in negative consequences for the user. One can see from the symptoms that an addiction framework will be used for the present work, although PSMU could also mean very different behavior such as cyberbullying online.

PSMU in terms of an addictive behavior (not officially recognized) has been linked to different health outcomes. Koc and Gulyagci [37] and Hong et al. [38] found that depressive symptoms positively predict Facebook addiction. Koc and Gulyagci [37] further identified anxiety and insomnia as positive predictors. Additionally, FoMO was identified as a strong predictor of (problematic) social media use [39, 40] and is also linked to both higher PSMU and lower meaning in life [41]. Furthermore, associations between PSMU and depression, anxiety, stress, higher cognitive failures [42] and poor sleep quality were found [4–6, 43–46]. Though most studies are cross-sectional, limiting causal interpretation, some longitudinal studies have been performed. One study found a bidirectional relationship between PSMU and depression and identified PSMU as predictor of insomnia, suicide related outcomes and ADHD symptoms [45]. PSMU could also lead to negative consequences such as low academic achievement, decrease in real life social participation, [47, 48] negative work-family balance, and decreased job performance [49].

Marino et al. [50] and Montag et al. [51, 52] showed moderate to high associations between PSU and PSMU, resulting from an overlap of both phenomena. See also other works [53, 54], showing robust overlap between PSU and distinct forms of social media overuse. While a lot of time is spent on social media, [1] not all smartphone use can be attributed to social media use, because smartphones also serve for gaming, browsing or video watching, instead, total screentime represents all uses. In the present study, total smartphone screentime was assessed as the original intention was to focus on smartphone gaming as well (see Procedure and Sample) and the smartphone serves as the platform for both social media engagement and gaming activities. Consequently, screentime serves as a comprehensive measure, reflecting both gaming and social media usage (but also a myriad of other activities including e-mail-checking, listening to music, etc.).

### Abstinence studies

Apart from assessing smartphone use and different outcomes, as mentioned above, assessing changes in outcomes due to not using the smartphone pose a possibility to infer about the causal direction of effects to answer questions such as that abstaining from smartphone and/or social media use results in less reported clinical symptoms, e.g. in the realm of depression or eating disorders. Several studies explored the effects of social media abstinence. Radtke et al. [55] found significant decreases in screen time during and after the intervention, mixed results on life satisfaction, decrease in anxiety and stress, improvement of sleep quality and mixed effects on FoMO and loneliness. However, the authors argue that different implementations of abstinence and measurements might account for the heterogeneity of findings. Another review by Fernandez et al. [56] found similar effects: increase in life satisfaction, affective well-being, decrease in perceived stress, and an increase in boredom, craving and time distortion.

Further studies – some with experimental designs – found a decrease in FoMO, increase in mental well-being and social connectedness, [57] and decreased depression and anxiety [58]. However, Vally and D’Souza [59] found a decrease in well-being, an increase in negative affect and loneliness during intervention and a nonsignificant increase in stress for the experimental group. Brailovskaia et al. [60] assessed if a full abstinence is necessary to see improvements in mental health or if a reduction of one hour per day would be enough. They found increased well-being and positive lifestyle changes in both experimental groups with stronger effects in the reduction group.

The effect sizes found in the mentioned studies are small to moderate with just few large effects.

Most of the aforementioned studies employed 7 days of abstinence with some exceptions where an abstinence of 14 days was implemented. Also, the foci of these studies were mainly on mental health variables like depression, anxiety, and FoMO. While these are key variables in the present study, another goal is to assess effects of social media abstinence on body image.

### Research questions and hypotheses

This study aims to assess the effect of a 14-day social media abstinence on different mental health and well-being factors using an experimental design. A follow-up assessment 14 days after the end of the intervention was implemented to assess stability of effects. A single 14-day follow-up was chosen due to economic reasons as retaining study participants is harder, the longer a study runs. Also, previous studies [55, 58, 60, 61] have realised different periods between end of intervention and follow-up (e.g. 48 hours, 4 days, 1 week, 1 month and 3 months), so using 14 days is somewhere in between, economically feasible and of the same length as the intervention period. Daily questionnaires were used to analyse changes during the intervention period.

The following hypotheses and research questions will be evaluated.

The hypotheses H1, H2.1 and H2.2 are based on baseline data collected before randomization into different groups.

*H1: In the overall sample, PSU is positively associated with reported total screentime, depression and anxiety symptom severity, and FoMO, respectively.*

Previous studies (but not all) showed a moderate positive association between PSU and objectively measured screentime [27, 36]. Although participants manually input screentime (total smartphone use, not just social media), similar low to moderate associations can be expected. The present study should also be able to replicate positive associations between PSU and depression and anxiety symptoms, and FoMO.

*H2.1: In the overall sample, screentime is positively but weakly associated with depression and anxiety scores, FoMO, and loneliness, respectively.*

*H2.2: In the overall sample, more screentime is negatively associated with body image.*

Many of these associations have not been shown with screentime, but with (problematic) smartphone use [37–40, 43]. This study did not assess (problematic) social media use. Instead, the variable of interest is screentime in association with different mental health outcomes. However, since a large amount of screentime also in the present participants is spent on social media, [1] it should be associated with the mentioned variables as well. Nonetheless, the correlations should be small, as Huang [62] showed in a meta-analysis that the time spent on social network sites is only weakly correlated with psychological wellbeing.

*H3: Screentime decreases in the experimental group.*

Since a good portion of screentime is spent on social media [1] an abstinence should reflect in overall decreased screentime. Total screentime was chosen as the measurement, because it reflects both time spent on social media and other smartphone activities.

*H4: Depression and anxiety scores, and PSU scores decrease in the social media abstinence group.*

Depression, anxiety, and PSU have been positively linked to problematic social media use [37, 38, 40, 43, 50]. So, reducing – or eliminating – social media should lead to decreasing symptoms. Additionally, previous abstinence studies showed decreased anxiety, stress and depression scores [55, 60].

*H5: Body image improves in the abstinence group.*

Body image is negatively associated with social media use, as exposure to idealized body types and social comparison in particular on visual driven social media platforms could lead to body dissatisfaction [7, 63, 64]. Although social media is not the only factor contributing to a negative body image, [65, 66] abstaining from it is likely to improve body image by reducing the exposure to social comparison.

*RQ1: How does FoMO change over time?*

Previous studies reported mixed results concerning changes in FoMO, [55, 57] possibly due to different intervention durations. Potentially, FoMO increases during the first few days of social media abstinence and then decreases once participants adapt to not using social media to check up on their friends. This study aims to provide insights into the changes over time during the abstinence phase by assessing FoMO daily and comparing different trends over time.

*RQ2: What is the impact of abstinence on loneliness?*

Several studies assessed the effect of social media abstinence on loneliness and found mixed results [55–57, 59]. Since loneliness was assessed daily, changes over the duration of abstinence can be detected and different trends can be compared.

*RQ3: Are the changes observed during abstinence stable after the intervention?*

Positive and negative changes due to abstinence from social media were already mentioned, but not much is known about the stability of these changes over time. Brailovskaia et al. [61] reported stable effects of changes after a 14-day gaming abstinence, however not much is known about stability after social media abstinence. Stability will be evaluated through change in scores between the end of intervention and the follow-up.

## Methods

### Procedure

**Fig. 1:**
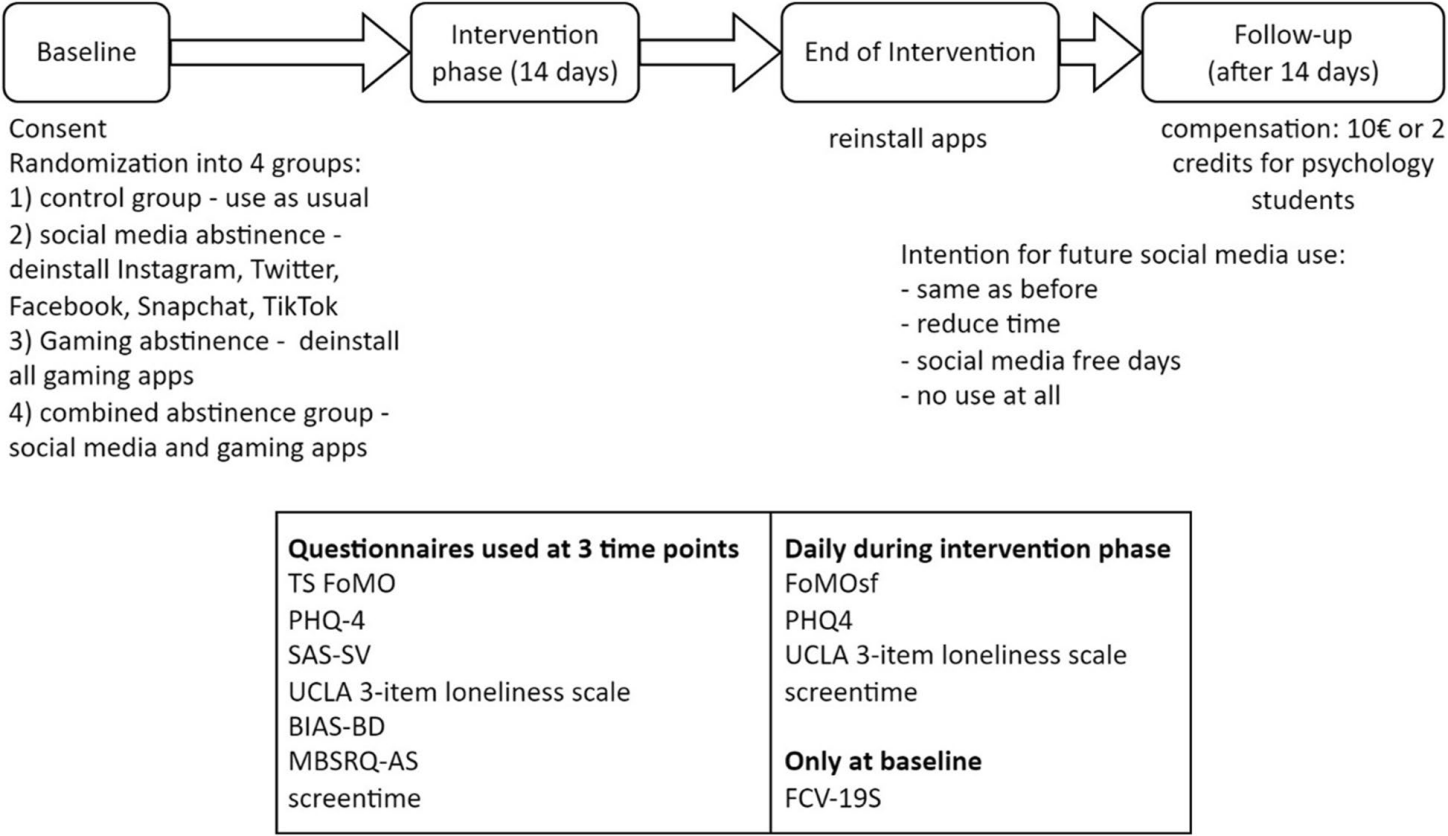
Schematic procedure. The procedure of the study is presented in the figure, the abbreviations for the included questionnaires are presented in the questionnaire section of this work

This study took place between October 2022 and February 2023. Participants were recruited via different university mailing lists, flyers posted around the university buildings, social media and eBay marketplace and underwent assessments outlined in Fig. 1. Inclusion criteria were: legal age (18+), good knowledge of the German language, and use of smartphone and social media. This online study was conducted using the SurveyCoder website, [67] with questionnaires administered at baseline, daily, end of intervention and at follow-up. Participants received a daily link to the website via email at 4 pm. After the baseline questionnaires, participants were randomized into four groups and received intervention instructions. Since no tracking apps were used, the deinstallation of apps was not monitored. Participants were allowed to use their smartphones as normal for all other purposes and were only instructed to deinstall apps from their smartphones (other devices were not mentioned in the instruction). At the end of the intervention, participants were allowed to reinstall apps and were asked how they intend to manage their future social media consumption.

Originally, comparisons between all groups were planned, however due to data cleaning steps (see Sample), only groups 1) and 2) were used for analyses in the main body of this manuscript.

### Sample

The initial sample compromised *N* = 196 participants who provided combined datasets (baseline, daily, end of intervention and follow-up). After exclusion of non-users of social media or gaming apps in the experimental groups, a sample of *n* = 165 participants was left. Since this sample consisted of 83.6% females and one focus of the study was the change in body image (which mainly shows effects for women), [68] only the female participants were analysed further.

From our view, this led to a too small group size for the gaming abstinence group to run robust statistics (*n* = 21). Since negative consequences due to gaming are mostly prevalent in men [69] and a small group size compared to the other groups can be problematic in analyses, this group was excluded from the main body of this manuscript (but see Supplement). The combined abstinence group (*n* = 31) was also excluded as this would have been relevant to provide insights in particular in comparison to both the distinct gaming and social media abstinence groups. But since the gaming abstinence group was excluded, it was decided to exclude this from the manuscript as well (again, for more information see Supplement).

Thus, the effective sample consisted of *n* = 86 female participants which were randomized into control group (*n* = 35) and social media abstinence group (*n* = 51). Most participants held A-level qualifications (64.0%) or university degrees (29.1%) and were currently enrolled at university (77.9%). Groups were comparable in terms of age (m_control_ = 23.17, s_control_ = 6.99; m_socmed_=24, s_socmed_=4.63; t(54.233) = -0.61, *p* = .54), education (majority have A-levels (63% in control group; 65% in social media abstinence group); followed by university degree (28% in control group; 29% in social media abstinence group), ${\chi }^{2}$(3) = 1.47, *p* = .69), and current occupational status (77% university students in control group; 78% university students in social media abstinence group; ${\chi }^{2}$(4) = 3.33, *p* = .50).

Analyses including excluded groups are presented in the Supplementary Materials 1 and 5 - 10.

### Questionnaires

#### Fear of missing out

Trait and online specific state FoMO was assessed using the TS FoMO scale [70] at baseline, end of the intervention and follow-up. Participants were asked to rate their agreement to 12 statements on a 5-point Likert scale (1 = “strong disagree”, 5 = “strong agree”). Mean scores for both subscales were computed and showed high internal consistency at all timepoints ($\alpha$ = 0.76 – 0.83 and $\alpha$ = 0.77 – 0.79, respectively). The German version as provided by Wegmann et al. [70] was used in the present work.

Daily FoMO was assessed using a single item question (FoMOsf; [71]): “Do you experience FoMO (the fear of missing out)?” Riordan et al. [71] proposed this single item assessment which showed good validity. Participants rated how much this applied to them on the current day on a 5-point Likert scale (1 = “no, not true of me”, 5 = “yes, extremely true of me”).

#### Problematic smartphone use

PSU was assessed using the Smartphone Addiction Scale – Short Version (SAS-SV; [72]) where participants rated their agreement with different statements concerning their smartphone use. These statements include difficulty concentrating, agitation in the absence of the smartphone, persistent preoccupation with the device, exceeding intended use duration, frequent checking behaviour, experiencing physical discomfort during use, and missed work obligations due to excessive smartphone use. Agreement was provided on a 6-point Likert scale ranging from 1 = “strongly disagree” to 6 = “strongly agree” and sum scores were used in analyses (higher values = more PSU). The scale showed high internal consistencies at all time points ($\alpha$ = 0.81 – 0.86). German version was used as in Haug et al. [73].

#### Depression and anxiety symptoms

Depression and anxiety symptoms were assessed using the 4-item Patient Health Questionnaire (PHQ-4; [74]). Participants were asked how often in the past seven days (for daily measurements: on the current day) they experienced different symptoms of depression or anxiety. Answers ranged from 0 = “no, not at all” to 3 = “nearly every day” (“nearly the whole day”) and were summed with higher values indicating more severe symptoms. The PHQ-4 demonstrated high internal consistency at all time points ($\alpha$ = 0.81 - 0.87).

#### Loneliness

How often participants experienced loneliness and isolation from others was assessed using the German version of the UCLA 3-item loneliness scale [75] as in Montag et al. [76]. Answers were given on a 3-point Likert scale (1 = “hardly ever”, 3 = “often”) and summed with higher values indicating higher loneliness. The scale demonstrated good internal consistency ($\alpha$ = 0.78 – 0.89).

#### Body image

Body Image Dissatisfaction (BID) was assessed using the BIAS-BD, [77] which presents two rows of schematic body figures ranging from 60% to 140% of the average BMI, separated for sex. Participants chose the figure best representing their actual and ideal body. Percentages were transformed into BMI equivalents and a BID score was computed as the difference between actual and ideal body size.

Further, the MBSRQ-AS [78] was used to measure different body image dimensions on 34 items: appearance evaluation (How content people are with their appearance), appearance orientation (How much attention people pay to their own appearance), body area satisfaction (How satisfied they are with different areas of their body), overweight preoccupation (How concerned they are with their weight and staying thin), and self-classified weight (How they would rate their own weight and how other people would rate their weight). Scores were summed for each dimension, and all showed good internal consistency with $\alpha =$ 0.66 – 0.92.

#### Screentime

Participants were asked to open the screentime feature on their smartphones and type the hours and minutes into the questionnaire. Values were converted into minutes for analysis. At baseline and follow-up, the screentime from the last seven days was averaged to represent the average daily screentime at baseline and follow-up, respectively. No differences were made between smartphone operating systems.

#### Fear of COVID-19

Fear of COVID-19 (FCV) was assessed at baseline using the FCV19S [79, 80] to use as a covariate in analyses. This was due to the study being conducted amid the COVID-19 pandemic (October 2022 to February 2023), allowing for the proper consideration of various pandemic-related constraints in the analyses. The FCV19S demonstrated a good internal consistency of $\alpha$ = 0.83. Participants were asked to rate seven statements concerning their fear of COVID-19 on a 5-point Likert scale from 1 = “strongly disagree” to 5 = “strongly agree”. Scores were summed for analysis and higher values indicated more Fear of COVID-19. The German version used herein was by Fatfouta and Rogoza [80].

#### Further questionnaires

The following questionnaires were assessed but not included in the main analyses. They are included in the supplement (see Supplementary Material): IPAQ (physical activity; [81]), PANAS – positive affect subscale, [82, 83] Perceived Stress Scale (PSS-4; [84, 85]) and satisfaction with life scale (SWLS; [86, 87]). The cited German versions were used for all scales.

### Data analysis

Data analysis was performed using R version 4.1.3 [88]. Apart from descriptive statistics at baseline, correlations were computed using Holm’s correction for *p*-values.

To model trends in outcome variables (PSU, FoMO, screentime, depression/anxiety, loneliness, body image), multilevel models were used. For the variables measured at three time points (baseline, end of intervention, follow-up), spline models were used with the knot point set to the end of intervention. This allows assessment of change between baseline values and end of intervention and between end and follow-up. Further, RQ3 can be answered using these models. For an explanation on spline models in the multilevel modelling framework, see Grimm et al. [89]. First, only the trend over time was modelled for the total sample (called model 1 for each outcome). Then, covariates were added (baseline FCV, PSU, and BID for MBSRQ-AS outcomes), and group differences were accounted for using dummy coded variables (0 = control, 1 = abstinence group; called model 2 for each outcome). Random intercepts were used for all models to allow for interindividual differences in values and where possible, random slopes were used to allow for interindividual differences in change over time. The R-packages *lme4* version 1.1.28 [90] and *lmerTest* version 3.1.3 [91] were used.

For daily measured outcomes, several multilevel models were computed. First, a linear trend over time for all experimental groups was evaluated. This model included baseline PSU, FCV, and the respective baseline value of each outcome. Then, the group variable was added to a separate model. To further assess changes over time in FoMO and loneliness (RQ1 and RQ2), different trends over time were assumed (quadratic, cubic) and models were compared using AIC, BIC and Likelihood Ratio Tests.

Datasets and analysis scripts are available at the Open Science Framework https://osf.io/qdp8r/.

The present study was approved by the university’s ethics committee (Ethics committee of University of Ulm) under application number 252/22.

## Results

Descriptive statistics at baseline are presented in Table 1. Due to randomization, no group differences were expected, and t-tests were non-significant.

**Table 1:** Descriptive statistics at baseline

	Total sample (*n* = 86)	Control group (*n* = 35)	Social media group (*n* = 51)	t-Test	
	Mean (s)	Mean (s)	Mean (s)		*p*
FCV	11.79 (4.39)	11.63 (3.90)	11.90 (4.73)	t(81.12) = 81.12	.77
Trait FoMO	3.22 (0.87)	3.32 (0.95)	3.15 (0.81)	t(65.21) = 0.87	.39
State FoMO	2.35 (0.79)	2.39 (0.77)	2.33 (0.82)	t(75.86) = 0.39	.69
PSU	32.01 (8.28)	31.94 (9.52)	32.06 (7.42)	t(61.06) = -0.06	.95
Depression/Anxiety	3.81 (2.97)	4.03 (2.83)	3.67 (3.08)	t(77.09) = 0.56	.58
Loneliness	4.92 (1.70)	5.20 (1.92)	4.72 (1.52)	t(61.85) = 1.22	.23
Appearance Evaluation	22.41 (5.93)	21.66 (5.21)	22.92 (6.38)	t(81.38) = -1.01	.32
Appearance Orientation	38.46 (8.23)	37.80 (8.31)	38.92 (8.23)	t(72.76) = --0.62	.54
Body Area Satisfaction	28.89 (5.84)	29.20 (4.72)	28.69 (6.53)	t(83.74) = 0.42	.67
Overweight Preoccupation	9.06 (3.44)	9.14 (3.52)	9.00 (3.42)	t(71.73) = 0.19	.85
Self-classified Weight	6.02 (1.15)	5.94 (1.00)	6.08 (1.25)	t(81.91) = -0.56	.58
Screentime (min)	244.93 (119.93)	243.12 (119.88)	246.18 (121.15)	t(73.73) = -0.11	.91
BID	2.64 (4.93)	2.42 (4.62)	2.79 (5.18)	t(78.33) = -0.35	.72

*FCV* Fear of COVID-19, *FoMO* Fear of Missing Out, *PSU* Problematic smartphone use, *BID* Body image dissatisfaction, *s* Standard deviation, *p* = unadjusted *p*-values (Bonferroni-adjusted *p*-values are all* p*_*adj*_ = 1)

### Correlations

Correlations at baseline are presented in Table 2 with Holm corrected *p*-values and 95% confidence intervals.

**Table 2:** Correlations at baseline

	1. Age	2	3	4	5	6	7	8	9	10	11	12	13
2. FCV	.08 [-.14, .28]												
3. Trait FoMO	-.22 [-.41, -.01]	.07 [-.14, .28]											
4. State FoMO	-.18 [-.38, .03]	.25 [.04, .44]	.49*** [.31, .64]										
5. PSU	-.21 [-.40, .00]	.16 [-.06, .36]	**.35** ^**a**^ [.15, .52]	.63*** [.48, .74]									
6. Depression/Anxiety	-.11 [-.31, .10]	.31 [.11, .49]	.1 [-.11, .31]	.18 [-.03, .38]	.26 [.05, .45]								
7. Loneliness	0 [-.21, .22]	.26 [.05, .44]	.36 [.16, .53]	.23 [.02, .42]	.34 [.13, .51]	.39* [.19, .56]							
8. Screentime (min)	-.16 [-.36, .06]	.25 [.05, .44]	-.02 [-.23, .19]	-.01 [-.23, .20]	.11 [-.10, .32]	.25 [.04, .44]	.16 [-.05, .36]						
9. BID	.09 [-.13, .29]	-.13 [-.33, .09]	.02 [-.19, .23]	0 [-.22, .21]	0 [-.21, .22]	.17 [-.04, .37]	.07 [-.14, .28]	.04 [-.17, .25]					
10. Appearance Evaluation	-.1 [-.30, .12]	-.32 [-.49, -.11]	-.22 [-.42, -.01]	-.20 [-.40, .01]	-.24 [-.43, -.03]	-.48*** [-.63, -.30]	-.55*** [-.68, -.39]	-.2 [-.39, .02]	-.32 [-.50, -.12]				
11. Appearance Orientation	-.31 [-.49, -.11]	0 [-.21, .21]	.23 [.02, .42]	.38* [.18, .54]	.29 [.08, .47]	-.05 [-.26, .17]	-.12 [-.32, .10]	.16 [-.05, .36]	-.09 [-.30, .12]	.14 [-.07, .34]			
12. Body Area Satisfaction	-.02 [-.23, .19]	-.31 [-.49, -.11]	-.2 [-.39, .02]	-.28 [-.46, -.07]	-.21 [-.41, -.00]	-.34 [-.52, -.14]	-.41** [-.57, -.22]	-.22 [-.42, -.01]	-.36 [-.53, -.16]	.81*** [.72, .87]	0 [-.22, .21]		
13. Overweight Preoccupation	-.22 [-.42, -.01]	-.04 [-.25, .17]	.15 [-.07, .35]	.19 [-.02, .39]	.05 [-.16, .26]	.09 [-.12, .30]	.11 [-.11, .31]	.03 [-.18, .24]	.47*** [.28, .62]	-.26 [-.45, -.05]	.33 [.12, .50]	-.31 [-.49, -.11]	
14. Self-classified Weight	.13 [-.09, .33]	-.18 [-.38, .03]	0 [-.21, .21]	-.05 [-.26, .16]	.09 [-.13, .30]	.14 [-.08, .34]	.19 [-.02, .39]	-.05 [-.26, .16]	.74*** [.63, .82]	-.36 [-.53, -.16]	-.15 [-.35, .06]	-.28 [-.47, -.08]	.43** [.23, .58]

*FCV* Fear of COVID-19, *FoMO* Fear of Missing Out, *PSU* Problematic smartphone use, *BID* Body Image Dissatisfaction. Holm corrected *p*-values. * *p* < .05, ** *p* < .01, *** *p* < .001. a: one-sided *p*-value < .05, 95% Confidence intervals

### Multilevel models

Results from models for outcome variables measured at three time points are presented in Table 3.

**Table 3:** Model outputs

			Depression/Anxiety	Screen time	PSU	Loneliness	State FoMO	trait FoMO	BID	Appearance Evaluation	Appearance Orientation	Overweight Preoccupation	Body Area Satisfaction	Self-classified Weight
			b (SE)	b (SE)	b (SE)	b (SE)	b (SE)	b (SE)	b (SE)	b (SE)	b (SE)	b (SE)	b (SE)	b (SE)
M1	fixed effects	Intercept	3.21*** (0.31)	223.00*** (12.48)	27.99*** (0.85)	4.25*** (0.18)	2.13*** (0.07)	2.69*** (115.56)	2.20*** (0.51)	23.39*** (0.65)	37.77*** (0.85)	8.77*** (0.38)	29.59*** (0.63)	5.92*** (0.11)
		T1	-0.60* (0.24)	-21.93 (11.38)	4.02*** (0.72)	-0.66*** (0.14)	-0.22*** (0.06)	-0.52*** (157.63)	-0.44 (0.25)	0.99** (0.36)	-0.70 (0.44)	-0.29 (0.20)	0.70 (0.37)	-0.10(0.06)
		T2	-0.03 (0.25)	9.08 (11.82)	1.46 (0.75)	0.16 (0.15)	-0.07 (0.06)	-0.08 (160.57)	0.01 (0.26)	-0.09 (0.38)	0.81 (0.46)	0.04 (0.21)	0.18 (0.38)	-0.01 (0.06)
	random effects	${\sigma }_{u\left(Intercept\right)}^{2}$	5.85 (2.42)	7840.00 (88.54)	50.46 (7.10)	1.93 (1.39)	0.48 (0.69)	0.58 (0.76)	21.65 (4.65)	31.31 (5.60)	60.47 (7.78)	10.23 (3.20)	25.58 (5.06)	0.97 (0.98)
		${\sigma }_{u\left(Time\right)}^{2}$			6.39 (2.53)	0.12 (0.35)	0.09 (0.30)	0.11 (0.33)	0.37 (0.61)		0.88 (0.94)	0.53 (0.73)	0.35 (0.60)	
	Fit	AIC	1116.2	3000.5	1667.1	858.86	429.63	534.1	1234.2	1387	1505.1	1124.9	1390.5	515.39
		BIC	1133.8	3018.1	1691.8	883.49	454.25	558.72	1258.9	1404.6	1529.7	1149.6	1415.1	532.98
M2	fixed effects	Intercept	-0.38 (1.22)	144.39** (50.16)	24.48*** (2.37)	2.05** (0.71)	0.70* (0.27)	2.12*** (0.37)	3.51 (2.30)	31.45*** (2.56)	32.18*** (3.78)	6.15*** (1.51)	37.74*** (2.48)	5.65*** (0.35)
		T1	-0.51 (0.38)	1.14 (17.78)	-2.91* (1.12)	-0.8*** (0.22)	-0.18 (0.09)	-0.41*** (0.11)	0.12 (0.38)	1.37* (0.57)	-1.17 (0.69)	-0.66* (0.32)	0.34 (0.58)	-0.08 (0.10)
		T2	-0.08 (0.39)	-6.13 (18.15)	-0.32 (1.15)	0.20 (0.22)	-0.10 (0.09)	-0.19 (0.11)	-0.05 (0.39)	-0.15 (0.58)	0.68 (0.70)	0.19 (0.32)	0.47 (0.59)	0.04 (0.10)
		Group	-0.58 (0.59)	-37.15 (25.12)	-1.86(1.70)	-0.28 (0.35)	-0.15 (0.13)	-0.37* (0.18)	-0.54 (1.04)	0.94 (1.17)	1.99 (1.70)	0.32 (0.69)	0.37 (1.17)	0.05 (0.16)
		FCV	0.23*** (0.06)	1.64 (1.29)	0.39* (0.17)	0.11** (0.03)	0.03* (0.01)	0.01 (0.02)	-0.14 (0.11)	-0.47*** (0.12)	-0.12 (0.18)	0.06 (0.07)	-0.43*** (0.12)	-0.03 (0.02)
		PSU	0.039 (0.03)	4.09 (2.44)		0.03 (0.02)	0.04*** (0.01)	0.02* (0.01)	0.02 (0.06)	-0.05 (0.07)	0.20* (0.10)	0.02 (0.04)	-0.07 (0.06)	0.01 (0.01)
		T1 * Group	-0.15 (0.49)	**-38.90** ^**a**^ ** (23.09)**	-1.87 (1.46)	0.23 (0.28)	-0.07 (0.12)	-0.19 (0.14)	**-0.95** ^**a**^ ** (0.50)**	-0.64 (0.74)	0.80 (0.89)	0.62 (0.41)	0.60 (0.75)	-0.03 (0.13)
		T2 * Group	0.08 (0.51)	25.36 (23.87)	-2.04 (1.52)	-0.07 (0.30)	0.05 (0.12)	0.18 (0.15)	0.08 (0.52)	0.10 (0.76)	0.22 (0.93)	-0.27 (0.43)	-0.52(0.78)	-0.08 (0.13)
		BID								-0.50*** (0.11)	-0.16 (0.16)	0.35*** (0.06)	-0.42*** (0.11)	0.15*** (0.01)
	random effects	${\sigma }_{u\left(Intercept\right)}^{2}$	4.77 (2.18)	7554 .00 (86.91)	48.92 (6.99)	1.58 (1.26)	0.27 (0.52)	0.52 (0.72)	21.96 (4.69)	22.79 (4.77)	57.35 (7.57)	7.90 (2.81)	18.74 (4.33)	0.39 (0.63)
		${\sigma }_{u\left(Time\right)}^{2}$			5.43 (2.33)	0.12 (0.35)	0.09 (0.30)	0.12 (0.34)	0.36 (0.60)		0.84 (0.92)	0.55 (0.74)	0.38 (0.62)	
	Fit	AIC	1103.4	2999.3	1664	848.77	512.56	533.98	1238.5	1368.5	1509.3	1107.8	1376.4	452.2
		BIC	1128	3023.9	1692.2	880.43	454.77	576.19	1280.7	1407.2	1555.1	1153.5	1422.2	490.9

T1 = Baseline vs. End of Intervention, T2 = End of intervention vs. follow-up, *PSU *Problematic smartphone use, *FoMO *Fear of Missing Out, *BID *Body image dissatisfaction, *AIC *Akaike Information Criterion, *BIC *Bayesian Information criterion, *FCV *Fear of COVID-19. Two-sided *p*-values: *p < .05, **p < .01, ***p < .001. One-sided *p*-values: ^a^ *p* < .05, b = estimated coefficient, SE = standard error. Group: 0 = control, 1 = social media abstinence

For depression and anxiety symptoms, model 1 with fixed slope showed a significant negative trend before the knot point (b = -0.60, t(160.76) = -2.52, *p* = .013), but no change afterwards. Model 2 showed no significant change over time or differences between groups.

**Fig. 2:**
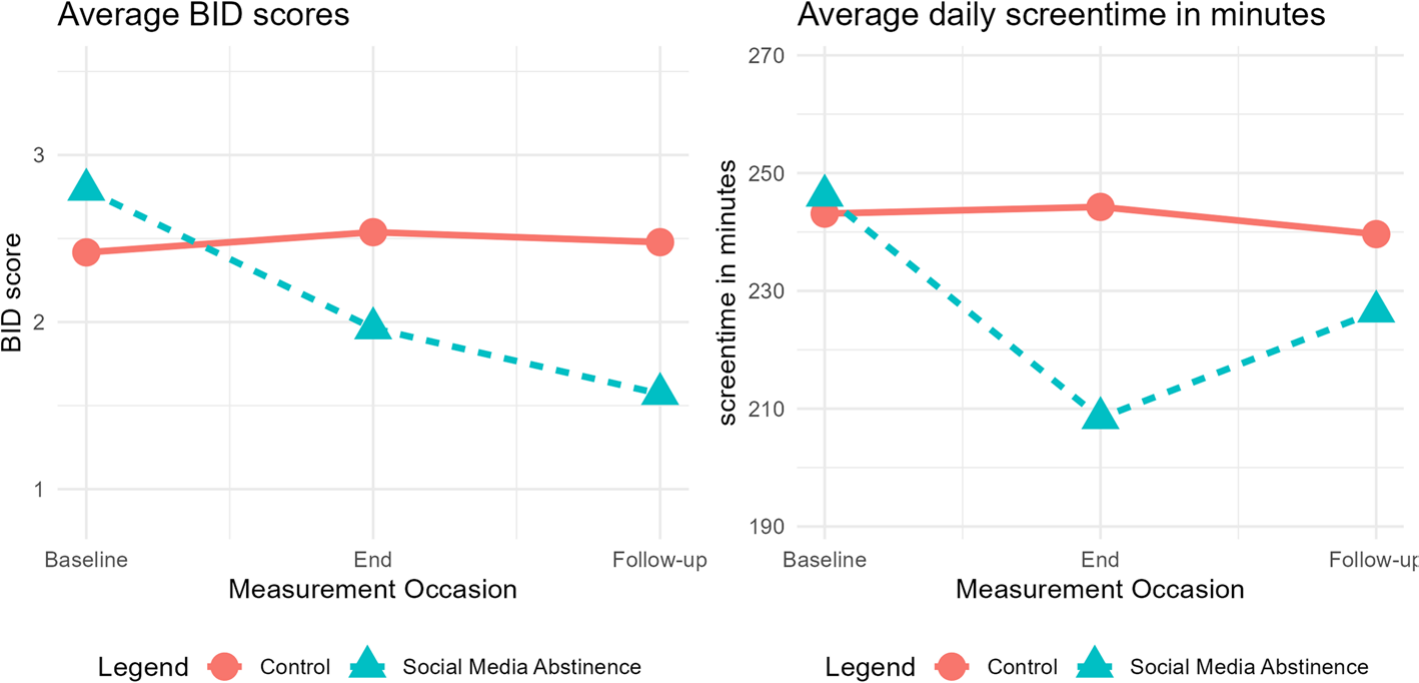
Plot of main results: changes in Body Image Dissatisfaction (BID) and average daily screentime

Model 1 for screentime showed nonsignificant changes across all time points. Upon incorporating covariates, no significant changes over time were observed for the control group. However, there was a significant difference in change between baseline and end of intervention between control group and abstinence group, as evidenced by one-sided testing (b = -38.905, t(159.8) = -1.685, *p* = .094/2 = .047). This indicates a decrease in screentime in the abstinence group. See Fig. 2 for a graphical representation of mean values in both groups.

Using PSU as outcome, model 1 showed a significant negative linear trend before the knot point (b = -4.023, t(150.54) = -5.583, *p* < .001) and a nonsignificant negative trend after the knot point. There was random variance for the trend over time. In model 2, only baseline FCV was added as covariate and had a significant influence on PSU. Further, the pre-knot negative trend was significant for the control group and no differences between groups were seen.

Model 1 for loneliness showed a significant negative linear change between baseline and end of intervention (b = -0.66, t(139.39) = -4.751, *p* < .001), indicating an overall decrease in loneliness during the intervention. In model 2, the negative linear change between baseline and end of intervention was significant for the control group and there were no group differences.

For state FoMO, model 1 showed a significant negative trend between baseline and end of intervention (b = -0.22, t(161.11) = -3.813,* p *< .001) and no change between end of intervention and follow-up. Model 2 showed no significant trend over time in the control group and no differences between groups.

There was a significant negative trend in model 1 for trait FoMO for the change between baseline and end of intervention (b = -0.52, t(157.63) = -7.30, *p* < .001) and no change afterwards. Model 2 showed a significant negative pre-knot point trend for the control group. This trend did not differ between groups, however the difference in values at the knot point (end of intervention) was significantly lower for the abstinence group.

The first model assessing trend over time showed a nonsignificant decrease in BID between baseline and end of intervention and no change afterwards. Model 2 showed no significant change over time for the control group. However, the difference in change between baseline and end of intervention was significant for one-sided testing (b = -0.95, t(139.64) = -1.900, *p* = .0595/2 = .029), indicating that BID values decreased more for the social media abstinence group compared to the control group. See Fig. 2 for a graphical representation of the mean values of both groups.

In model 1, appearance evaluation showed a significant increase between baseline and end of intervention (b = 0.988, t(161.02) = 2.736, *p* < .001) and no change afterwards. In model 2, the positive change between baseline and end of intervention was only significant for the control group.

Overweight preoccupation showed no change over time in model 1. Upon adding covariates and a group variable, there was a significant negative trend for the change between baseline and end of intervention in the control group. No group differences were found.

Model 1 revealed an almost significant increase in body area satisfaction during the intervention (b = 0.698, t(136.24) = 1.899, *p* = .0597) and no change afterwards. However, this trend was not significant in model 2 with covariates and group variable, nor was there a difference between groups.

No effect of either time or group could be identified for self-classified weight and appearance orientation.

### Daily data models

For the daily data models, different trends were modelled for each variable. As covariates in all models the respective values at baseline were used as well as baseline FCV19, PSU, and BID. Results are provided in Table 4.

**Table 4:** Daily data models

Outcome variable: Screentime																																				
	Fixed Effects																												Random effects			Fit				
	Intercept				Day	Day^2^						Group		Baseline PSU	Baseline FCV			Baseline BID		Baseline screentime			Day * Group	Day^2^ * Group					${\sigma }_{u\left(Intercept\right)}^{2} ({\sigma }_{u\left(Intercept\right)})$			AIC			BIC	
M1	18.01 (29.31)				0.51 (0.62)									1.60* (0.75)	-0.45 (1.46)			-2.01 (1.24)		0.69*** (0.05)									2551 (50.51)			13408			13448	
M2	11.48 (29.66)				3.79 (2.33)	-0.25(0.17)								1.61* (0.75)	-0.45 (1.46)			-2.01 (1.24)		0.69*** (0.05)									2556 (50.56)			13408			13453	
M3	22.94 (30.52)				0.14 (0.99)							-8.94 (14.87)		1.61* (0.75)	-0.44 (1.46)			-1.99 (1.25)		0.69*** (0.05)			0.61 (1.27)						2582 (50.82)			13412			13462	
M4	25.71 (31.32)				-1.33 (3.69)	0.11 (0.28)						-24.63 (17.37)		1.61* (0.75)	-0.43 (1.47)			-1.98 (1.25)		0.69*** (0.05)			8.51 (4.75)	-0.61 (0.35)					2591 (50.9)			13411			13471	
Outcome variable: Loneliness																																				
	Fixed Effects																													Random Effects		Fit				
	Intercept				Day			Day^2^		Day^3^		Group	Baseline PSU		Baseline FCV			Baseline BID		Baseline loneliness			Day * Group		Day^2^ * Group			Day^3^ * Group		${\sigma }_{u\left(Intercept\right)}^{2} ({\sigma }_{u\left(Intercept\right)})$		AIC			BIC	
M1	1.64** (0.51)				-0.01 (0.01)								-0.01 (0.01)		0.09*** (0.03)			0.03 (0.02)		0.37*** (0.07)										0.86 (0.93)		3601.2			3641.4	
M2	1.71** (0.51)				-0.04 (0.03)			0.01 (0.01)					-0.01 (0.01)		0.09*** (0.03)			0.03 (0.02)		0.37*** (0.07)										0.86 (0.93)		3601.7			3646.9	
M3	1.85*** (0.52)				-0.20** (0.07)			0.04** (0.01)		0.01** (0.01)			-0.01 (0.01)		0.10*** (0.03)			0.03 (0.02)		0.36*** (0.07)										0.86 (0.93)		3596.9			3647.2	
M4	1.45** (0.52)				-0.01 (0.01)							0.31 (0.24)	-0.01 (0.01)		0.09*** (0.02)			0.03 (0.02)		0.39*** (0.07)			0.01 (0.02)							0.83 (0.91)		3601			3651.3	
M5	1.48** (0.53)				-0.03 (0.05)			0.00 (0.00)				0.38 (0.27)	-0.01 (0.01)		0.09*** (0.03)			0.03 (0.02)		0.39*** (0.07)			-0.02 (0.06)		0.00 (0.00)					0.83 (0.91)		3603.1			3663.5	
M6	1.73** (0.54)				-0.33** (0.11)			0.06** (0.02)		0.01** (0.01)		0.20 (0.29)	-0.02 (0.01)		0.09*** (0.03)			0.03 (0.02)		0.38*** (0.07)			0.20 (0.14)		-0.04 (0.03)			0.01 (0.01)		0.83 (0.91)		3597.2			3667.6	
Outcome variable: Depression and anxiety																																				
		Fixed effects																													Random effects		Fit			
		Intercept		Day								Group	Baseline PSU			Baseline FCV	Baseline BID		Baseline PHQ			Day * Group									${\sigma }_{u\left(Intercept\right)}^{2} ({\sigma }_{u\left(Intercept\right)})$		AIC			BIC
M1		-0.51 (0.81)		0.01 (0.01)									-0.01 (0.02)			0.13** (0.04)	0.03 (0.04)		0.43*** (0.06)												2.26 (1.50)		4689			4729.3
M2		-0.01 (0.01)		-0.04 (0.02)								-0.14 (0.39)	-0.01 (0.02)			0.13** (0.04)	0.03 (0.04)		0.43*** (0.06)			0.06* (0.03)									2.28 (1.51)		4686.5			4736.8
Outcome variable: FoMO																																				
		Fixed effects																													Random effects		Fit			
		Intercept	Day				Day^2^		Day^3^		Group			Baseline PSU		Baseline FCV	Baseline BID		Baseline trait FoMO		Baseline state FoMO	Day * Group				Day^2^ * Group	Day^3^ * Group				${\sigma }_{u\left(Intercept\right)}^{2} ({\sigma }_{u\left(Intercept\right)})$		AIC	BIC		
M1		0.29 (0.37)	-0.02*** (0.01)											0.00 (0.01)		0.05** (0.02)	0.03* (0.01)		0.23* (0.09)		0.18 (0.12)										0.36 (0.60)		2880.6	2925.9		
M2		0.36 (0.37)	-0.05* (0.02)				0.01 (0.01)							0.00 (0.01)		0.05** (0.02)	0.03* (0.01)		0.23* (0.09)		0.17 (0.12)										0.36 (0.60)		2879.8	2930.1		
M3		0.54 (0.37)	-0.25*** (0.05)				0.04*** (0.01)		0.01*** (0.01)					0.00 (0.01)		0.05** (0.02)	0.03* (0.01)		0.23* (0.09)		0.17 (0.12)										0.36 (0.60)		2862.6	2918		
M4		0.20 (0.38)	-0.02* (0.01)								0.14 (0.16)			0.00 (0.01)		0.05** (0.02)	0.03* (0.01)		0.24* (0.09)		0.18 (0.12)	0.00 (0.01)									0.36 (0.60)		2883.2	2938.5		
M5		0.21 (0.39)	-0.02 (0.03)				0.00 (0.00)				0.25 (0.18)			0.00 (0.01)		0.05** (0.02)	0.03* (0.01)		0.24* (0.09)		0.18 (0.12)	-0.05 (0.04)				0.00 (0.00)					0.36 (0.60)		2882.7	2948.1		
M6		0.33 (0.39)	-0.17* (0.08)				0.03* (0.01)		0.01* (0.01)		0.33 (0.20)			0.00 (0.01)		0.05** (0.02)	0.03* (0.01)		0.24* (0.09)		0.17 (0.12)	-0.14 (0.10)				0.02 (0.02)	0.00 (0.00)				0.36 (0.60)		2866.4	2941.9		

*AIC* Akaike Information Criterion, *BIC* Bayesian Information Criterion, *FCV* Fear of COVID19, *BID* Body image dissatisfaction, *PSU* problematic smartphone use, *FoMO *Fear of Missing Out, *PHQ *Depression and anxiety symptom severity, Group: 0 = control, 1 = social media abstinence. *p < .05, **p < .01, ***p < .001

For the total sample, linear (model 1) or quadratic (model 2) trend over time for screentime could not be found. Upon adding the group variable in the quadratic trend model (model 4), the interaction term for linear trend and abstinence group was almost significant (b = 8.56, t(1046.18) = 1.800, *p* = .072), as well as the interaction between the quadratic trend and the abstinence group (b = -0.61, t(1045.28) = -1.727,* p* = .084). This indicates a different change in screentime for the social media abstinence group than observed in the control group.

Linear (model 1) and quadratic (model 2) trends for changes in loneliness were not supported for the total sample. Model 3 assumed a cubic trend and found significant results for the linear, quadratic and cubic parts of the trend. Model comparisons between three models identified model 3 as the best fitting model (AIC = 3596.9, BIC = 3647.2, M2 vs. M3: ${\chi }^{2}$(1) = 6.78, *p* < .01).

Models 4 (linear trend and group) and 5 (quadratic trend and group) showed no trends over time nor group differences. Model 6 – including a cubic trend – showed significant linear, quadratic and cubic trends for the control group and no differences between groups. This model fit the data best (AIC = 3597.2, BIC = 3667.6, M5 vs. M6: ${\chi }^{2}$(2) = 9.92,* p* < .01). However, there was no significant difference between models with and without the group variable (M3 vs. M6: ${\chi }^{2}$(4) = 7.6911, *p *= .1036).

Model 1 showed no significant linear trend over time in depression and anxiety symptoms. Model 2 included the linear trend over time and the group variable and showed an almost significant negative change for the control group (b = -0.04, t(1048.51) = -1.834, *p *= .067) and a significant interaction between daily change and the abstinence group (b = 0.06, t(1046.40) = 2.429, *p *< .05), indicating different changes over time between both groups.

Model 1 found a significant negative linear trend with an average 0.0195 decrease per day for FoMO. Model 2 included a quadratic trend as well as the linear trend and found the linear trend to be significant (b = -0.054, t(1043) = -2.506,* p* < .05). Model 3 assumed a cubic trend and found this to be significant. Model comparisons identified model 3 as best fitting (AIC = 2862.6, BIC = 2918.0, M2 vs. M3: ${\chi }^{2}$(1) = 19.116, *p* < .001).

Model 4 found a significant negative linear trend for the control group and no group differences. Model 5 (quadratic trend) found no significant linear or quadratic trend for either group. Model 6 (cubic trend) found a significant linear, quadratic and cubic trend for the control group, but no difference between the groups. Model 6 was identified as the best fitting model containing the group variable (AIC = 2866.4, BIC = 2941.9, M5 vs. M6: ${\chi }^{2}$(2) = 20.22, *p* < .001), however the fit was not significantly different from model 3 (M3 vs. M6: ${\chi }^{2}$(4) = 4.1824, *p* = .3819).

## Discussion

The aim of this study was to evaluate the impact of a 14-day social media abstinence on different mental health and well-being variables and body image. Results are discussed below.

### Associations between variables

The study’s findings align with the expectations outlined in H1. PSU demonstrated a weak positive association with screentime, although it was not statistically significant, which is consistent with prior research [27, 36]. This supports the notion that self-reported PSU and screentime is not necessarily the same construct and that screentime is not an appropriate measure for PSU. Furthermore, different uses and motives for smartphone use can explain why some people have high screentime but low PSU.

Motives can be evaluated using the CIUT [31]. Though literature on associations between smartphone use motives and screentime is not exhaustive, studies have found that motives like mood regulation and enjoyment are positively associated with PSU, whereas information seeking and socializing are less likely to have an influence on addictive behaviour in the realm of smartphones [92, 93]. Additional motives for use were distress tolerance and mindfulness, [94] FoMO, [95, 96] and boredom proneness [96, 97].

PSU was assumed to be positively associated with depression and anxiety symptom severity (H1) and a moderate association (albeit not significant for this sample) has been found. Again, this is consistent with previous literature [24–27].

The hypothesized association between PSU and state FoMO was highly positive whereas the association with trait FoMO was moderately positive, supporting H1. Both can be interpreted as people experiencing more PSU symptoms also experience more FoMO. Again, these results are in accordance with previous studies identifying FoMO as a correlate of PSU [25]. Furthermore, according to the I-PACE model [33, 34] trait FoMO can be seen as a core characteristic impacting how certain situations are received and responded to, thus, contributing to the development of PSU (please note that due to the overlap with neuroticism, it might be also seen as a trait; [98]).

Positive but weak associations were found for screentime and depression/anxiety symptom severity, FoMO, and loneliness, supporting H2.1. All associations are low (to moderate for screentime and depression/anxiety) and not significant in the present sample. This was expected, as Huang [62] reported very small associations between time spent on social network sites and mental health variables. There are different uses of smartphones that can be unproblematic but lead to high screentimes (e.g attending online meetings or using the phone to study). This should be controlled for in future studies.

Hypothesis H2.2 assumed a negative correlation between screentime and body image. This hypothesis is supported only descriptively, as no correlation is significant. Screentime showed weak negative associations with appearance evaluation and body area satisfaction, and positive associations with appearance orientation (see that also using objective screentime-measures, a recent work by Rozgonjuk et al. [64] established links between longer smartphone use and higher body dissatisfaction; in this work also patients with eating disorders were investigated). The present findings suggest that individuals who spend more time on their smartphones are a little more appearance oriented and a little less satisfied with their bodies. However, it is crucial to note that screentime and exposure to online media are not the sole factors influencing BID [65, 66]. Studies found that the type of screentime influences development of BID, at least for TV or computers [99–101]. Specifically, computer use for leisure activities was positively associated with BID whereas computer use for homework showed negative associations with BID [99]. Hrafnkelsdottir et al. [100] found positive correlations between gaming, TV/DVD/internet watching and BID and low correlations between BID and online communication. This suggests that different uses of smartphones and social media might have different impacts on body image. An assessment of motives of use could provide further insight.

### Changes over time

An overall decrease in screentime during the intervention, especially for the abstinence group was found, supporting hypothesis H3. Since a large portion of screentime is spent on social media, [1] abstinence from selected applications should be reflected in overall decreased screentime. These results align with previous abstinence studies which also reported decreased screentime [55, 57]. However, on a day-to-day basis during the intervention, no significant changes in screentime were found. This could be attributed to fluctuating screentimes or compensatory behaviour, such as switching to other apps to fill the time.

Depression and anxiety scores decreased when assessing the total sample but there was no change nor difference between groups when considering the group variable. Therefore, H4 is not supported for depression and anxiety. Contrary to the hypothesis, daily models showed a decrease in the control group and an increase in the experimental group (please note that these observations are on a descriptive level only and changes were not pronounced). However, according to the CIUT, [31] smartphones and by extension social media can act as a coping mechanism and as an escape to handle negative emotions and daily hassles. If this outlet is unavailable, symptoms of depression and anxiety might increase (we are not of the opinion though that social media use should be seen as an effective way to deal with one's own problems and it is unclear how long lasting the effect around depression and anxiety would be). Motives of use are often evaluated in gaming research and escapism was identified as a strong predictor for gaming time (and gaming disorder, [102]), highlighting the tendency of dealing with negative emotions by escaping into an online world [103].

Additional analyses were conducted to examine the relationship between changes in depression and anxiety scores and baseline PSU, across groups (total sample). The results indicated a weak negative correlation, suggesting that, across groups, individuals with higher baseline PSU scores experienced more decrease in depression and anxiety scores compared to those with lower PSU scores. Since there was only a minimal difference in baseline PSU scores between the experimental groups (see Table 1), the association between baseline PSU values and change in depression and anxiety scores cannot be the reason for the different trends over time measured in the depression and anxiety scores.

However, when baseline depression and anxiety scores were correlated with changes in anxiety and depression scores, a moderate negative correlation emerged. The control group displayed slightly higher baseline scores than the abstinence group, although this difference was not statistically significant. This provides a possible explanation to the reduction in depression and anxiety scores in the control group compared to the abstinence group.

For PSU, an overall decrease was found during and after the intervention. However, there were no differences between groups. Possibly, the study attracted individuals seeking to change their social media habits as it was advertised as an abstinence study. Intention towards future social media use was assessed at the end of intervention and follow-up with most participants expressing a desire to reduce their social media time (end of intervention: 57% in control group, 49% in abstinence group; follow-up: 48% in control group, 66% in abstinence group). Since there was no big group difference in the number of participants with this answer, controls possibly intended to reduce their social media consumption even before their study participation and changed their behaviour, thus experiencing less PSU.

Additionally, PSU is not synonymous with social media use. Though studies found a strong positive association between PSU and PSMU, [50] PSU can develop through other smartphone uses than social media. Plus, participants were asked to abstain only from selected social media but were able to freely use their phones for other uses.

Body image was assessed using different variables. Appearance orientation and self-classified weight showed no changes over time or between groups. There was an overall increase in appearance evaluation and body area satisfaction due to the intervention, but no differences between groups. Overweight preoccupation decreased for the control group and there was no difference in changes between groups.

The BID values decreased significantly more in the abstinence group than in the control group, suggesting that taking a break from exposure on social media is helpful in decreasing BID. Overall, hypothesis H5, suggesting social media abstinence improves body image satisfaction and decreases dissatisfaction, was partially supported. However, the effect is small, as social media is not the only factor influencing body image [65, 66]. Social comparison occurs not only on social media but in real-life interactions and through other media like TV or magazines.

Daily change in FoMO (RQ1) was best modelled using a cubic trend. Nevertheless, there were no group differences, suggesting day-to-day fluctuation in FoMO regardless of whether social media apps were used or not. Previous studies found mixed outcomes regarding intervention on FoMO, [55, 57] but only assessed it for 7 days. Since FoMO fluctuates, assessing changes over a longer period of time offers a more comprehensive dataset for fitting appropriate models.

Trait and state FoMO decreased during intervention in both groups and remained stable afterwards. The absence of group differences can be attributed to individuals using their phones for different uses that do not necessarily influence FoMO. Elhai et al. [104] found that FoMO is more associated with non-social smartphone use like entertainment, news and relaxation compared to social smartphone use. Though PSU was positively related to both trait and state FoMO, screentime was not associated with either. This suggests that simply abstaining from social media may not lead to reduced FoMO, at least not in the here investigated time interval. Furthermore, since traits are considered relatively stable constructs, [105] it is debatable if a change in trait FoMO can be expected. Trait FoMO can be also conceptualized as dispositional factor in the I-PACE model [33, 34] and is a stable influence on the development of PSU. Since dispositional factors are not expected to change strongly – especially not in a short time frame – the observed change was more likely an artifact in data.

A cubic trend was also the best way to model daily changes in loneliness (RQ2), though there were no significant group differences. Both groups experienced a decrease in loneliness during the intervention and no change afterwards. This suggests that overall, loneliness decreased but due to factors other than not using social media apps. This study did not assess other life events, making it challenging to explain this change fully. Furthermore, previous studies found mixed effects of abstinence on loneliness [55–57, 59]. Moreover, there are different motives for social media use and not all are related to social interaction. These results can also be interpreted in the context of the uses and gratification theory [106] as the smartphone can be used to fulfil individuals needs such as representation, maintaining social networks, receiving online support, relaxing, or escaping from pressures [107]. Not all motives are related to loneliness.

The majority of spline models did not show significant changes after the intervention, indicating stability in the effects and addressing RQ3. Specifically, this applies to changes in BID and screentime, where differences between the experimental and control groups were seen. For the other variables, there were no group differences, but the changes between baseline and end of intervention measurements suggested an overall decrease (depression and anxiety, PSU, overweight preoccupation, state and trait FoMO, loneliness) or increase (appearance evaluation, body area satisfaction) and no change afterwards. However, these changes apply to both groups, meaning the control group changed as well and abstinence was not the sole reason for change, but maybe the intention to reduce consumption was.

### Contribution and limitations

The present study provides novel insights into the relationships between social media use and mental health and well-being. Notably, this study used an experimental design to implement a 14-day intervention and conducted a follow-up assessment 14 days after this intervention. Furthermore, this work focussed on body image and its changes over time. This can provide information for future studies or intervention designs as it shows that body image dissatisfaction can be decreased by not using social media for 14 days. The experimental approach adds depth to the understanding of the impact of social media on body image, a topic that has primarily been explored through correlative studies. The present results can also provide a first basis for inventing and implementing interventions in the realm of eating disorders or body schema disorders, as it shows that abstaining from social media might improve body satisfaction (replication of the present findings is of importance). But: As there is currently no consensus or official diagnosis for PSU and also against the limitations mentioned below, authors refrain from proposing clinical implications based on reduced screentime during intervention at the moment.

Additionally, previous studies modelled FoMO as linear change over time and often only assessed one week of change. The present study provides more detailed insight into daily FoMO as well as daily loneliness changes and found that both can be best represented using a cubic trend.

There are several limitations. First, the original study design intended to include four groups for comparison, but due to a high percentage of women in the sample, only female participants were analysed in the main manuscript, leading to exclusion of the gaming disorder groups. Consequently, small sample sizes were used for analyses, resulting in low statistical power. While some results were directionally clear, they did not reach statistical significance in the present work. Second, PSMU was not assessed alongside PSU, which should be considered in future studies. Screentime was not objectively measured but participants manually input the information from their screentime feature (hence we have an indirect objective screentime measure, which might be prone to transfer error though but was checked for plausability). In further studies, an objective measurement could be implemented by either using tracking apps – and thus validating if participants use social media apps – or asking for screenshots of the screentime feature. Measurement of total screentime and PSU were chosen as the original intention was to include the gaming and combined abstinence groups. In that case, both screentime and PSU would be acceptable measurements for all groups, as gaming and social media use can be reflected in total screentime and can both lead to symptoms of PSU.

Aside from assessing PSMU and using an objective measure for future studies it is suggested to assess motives and uses for individual’s smartphone use because this could provide further insight into why outcomes change for some participants but not for all. This could also aid in developing more nuanced interventions that properly fit a person’s needs. Lastly, different groups with different levels of abstinence could be realized. This has previously been done by Brailovskaia et al. [60] for general smartphone use.

## Conclusion

Using a longitudinal and experimental approach to a 14-day social media abstinence, the present study was able to show significant decreases in BID and screentime due to abstinence. Further, mental well-being factors were evaluated and showed improvement over time but did not differ between groups. Using daily assessments of FoMO and loneliness, cubic trends were identified as the best way to model fluctuation in these variables. These findings provide valuable insights into the complex dynamics of social media use and its impact on mental health and well-being and can provide information to plan future interventions addressing social media/smartphone use or body image related disorders.

### Supplementary Information

**Supplementary Material 1.** 

## Data Availability

Datasets and analysis scripts are available at the Open Science Framework https://osf.io/qdp8r/.

## Abbreviations

AIC: Akaike Information criterion
BIC: Bayesian information criterion
BID: Body image dissatisfaction
BMI: Body mass index
CIUT: Compensatory Internet Use Theory
FCV: Fear of COVID-19
FoMO: Fear of Missing Out
H: Hypothesis
PANAS: Positive affect negative affect scale
PHQ-4: Patient health questionnaire 4
PSMU: Problematic social media use
PSS: Perceived stress scale
PSU: Problematic smartphone use
RQ: Research question
SAS-SV: Smartphone Addiction Scale – short version
SWLS: Satisfaction with Life scale
TS FoMO: Trait State FoMO scale

## References

[1] We Are Social Ldt. We Are Social UK. 2023. Cited 2023 Mar 15. The Changing World of Digital In 2023. Available from: https://wearesocial.com/uk/blog/2023/01/the-changing-world-of-digital-in-2023/.

[2] Forbes Health. 2023. Cited 2023 Mar 17. Mental Health Statistics. Available from: https://www.forbes.com/health/mind/mental-health-statistics/.

[3] Twenge JM, Joiner TE, Rogers ML, Martin GN (2018). Increases in Depressive Symptoms, Suicide-Related Outcomes, and Suicide Rates Among U.S. Adolescents After 2010 and Links to Increased New Media Screen Time. Clin Psychol Sci.

[4] Bashir H, Bhat S (2016). Effects of social media on mental health: a review. Int J Indian Psychol.

[5] Dhir A, Yossatorn Y, Kaur P, Chen S (2018). Online social media fatigue and psychological wellbeing—A study of compulsive use, fear of missing out, fatigue, anxiety and depression. Int J Inf Manag.

[6] Karim F, Oyewande AA, Abdalla LF, Ehsanullah RC, Khan S. Social Media Use and Its Connection to Mental Health: A Systematic Review. Cureus. 2020;12(6). Cited 2022 Jun 28. Available from: https://www.cureus.com/articles/31508-social-media-use-and-its-connection-to-mental-health-a-systematic-review. 10.7759/cureus.8627 PMC7364393 32685296

[7] Fardouly J, Vartanian LR (2016). Social media and body image concerns: current research and future directions. Curr Opin Psychol.

[8] Grimaldi-Puyana M, Fernández-Batanero JM, Fennell C, Sañudo B (2020). Associations of objectively-assessed smartphone use with physical activity, sedentary behavior, mood, and sleep quality in young adults: a cross-sectional study. Int J Environ Res Public Health..

[9] Lin ML, Wang WY, Liao CC, Luo YJ, Kao CC (2020). Examining thevel among University Students in Taiwan. Healthcare.

[10] Shimoga SV, Erlyana E, Rebello V (2019). Associations of social media use with physical activity and sleep adequacy among adolescents: cross-sectional survey. J Med Internet Res.

[11] Billieux J, Maurage P, Lopez-Fernandez O, Kuss DJ, Griffiths MD (2015). Can disordered mobile phone use be considered a behavioral addiction? An update on current evidence and a comprehensive model for future research. Curr Addict Rep.

[12] Panova T, Carbonell X (2018). Is smartphone addiction really an addiction?. J Behav Addict.

[13] Rod NH, Dissing AS, Clark A, Gerds TA, Lund R (2018). Overnight smartphone use: A new public health challenge? A novel study design based on high-resolution smartphone data. PLOS One.

[14] Sohn SY, Rees P, Wildridge B, Kalk NJ, Carter B (2019). Prevalence of problematic smartphone usage and associated mental health outcomes amongst children and young people: a systematic review, meta-analysis and GRADE of the evidence. BMC Psychiatry.

[15] Yang J, Fu X, Liao X, Li Y (2020). Association of problematic smartphone use with poor sleep quality, depression, and anxiety: a systematic review and meta-analysis. Psychiatry Res.

[16] Derks D, Bakker AB (2014). Smartphone use, work-home interference, and burnout: a diary study on the role of recovery. Appl Psychol.

[17] Hawi NS, Samaha M (2016). To excel or not to excel: Strong evidence on the adverse effect of smartphone addiction on academic performance. Comput Educ..

[18] Lee W Jun, Shin S. A Comparative Study Of Smartphone Addiction Drivers’ Effect On Work Performance In The U.S. And Korea. J Appl Bus Res. 2016;32(2):507–16.

[19] Samaha M. Relationships among smartphone addiction, stress, academic performance, and satisfaction with life. Comput Hum Behav. 2016;321–5.

[20] Kim SY, Koo SJ (2016). Effect of duration of smartphone use on muscle fatigue and pain caused by forward head posture in adults. J Phys Ther Sci.

[21] Park J, Kim J, Kim J, Kim K, Kim N, Choi I, et al. The effects of heavy smartphone use on the cervical angle, pain threshold of neck muscles and depression. Adv Sci Technol Lett. 2015;91:12–7.

[22] McCrann S, Loughman J, Butler JS, Paudel N, Flitcroft DI. Smartphone use as a possible risk factor for myopia. Clin Exp Optom. 2021;104(1). Cited 2021 May 24. Available from: https://onlinelibrary.wiley.com/doi/abs/10.1111/cxo.13092. 10.1111/cxo.13092 32452059

[23] Wang J, Li M, Zhu D, Cao Y (2020). Smartphone overuse and visual impairment in children and young adults: systematic review and meta-analysis. J Med Internet Re..

[24] Demirci K, Akgönül M, Akpinar A (2015). Relationship of smartphone use severity with sleep quality, depression, and anxiety in university students. J Behav Addict.

[25] Elhai JD, Levine JC, Dvorak RD, Hall BJ. Fear of missing out, need for touch, anxiety and depression are related to problematic smartphone use. Comput Hum Behav. 2016;63:509–16.

[26] Elhai JD, Dvorak RD, Levine JC, Hall BJ (2017). Problematic smartphone use: a conceptual overview and systematic review of relations with anxiety and depression psychopathology. J Affect Disord.

[27] Rozgonjuk D, Levine JC, Hall BJ, Elhai JD (2018). The association between problematic smartphone use, depression and anxiety symptom severity, and objectively measured smartphone use over one week. Comput Hum Behav.

[28] Thomée S, Härenstam A, Hagberg M (2011). Mobile phone use and stress, sleep disturbances, and symptoms of depression among young adults - a prospective cohort study. BMC Public Health.

[29] Wolniewicz CA, Tiamiyu MF, Weeks JW, Elhai JD (2018). Problematic smartphone use and relations with negative affect, fear of missing out, and fear of negative and positive evaluation. Psychiatry Res.

[30] Yuan G, Elhai JD, Hall BJ (2021). The influence of depressive symptoms and fear of missing out on severity of problematic smartphone use and Internet gaming disorder among Chinese young adults: A three-wave mediation model. Addict Behav.

[31] Kardefelt-Winther D (2014). A conceptual and methodological critique of internet addiction research: towards a model of compensatory internet use. Comput Hum Behav.

[32] Montag C, Marciano L, Schulz PJ, Becker B (2023). Unlocking the brain secrets of social media through neuroscience. Trends Cogn Sci..

[33] Brand M, Wegmann E, Stark R, Müller A, Wölfling K, Robbins TW (2019). The Interaction of Person-Affect-Cognition-Execution (I-PACE) model for addictive behaviors: Update, generalization to addictive behaviors beyond internet-use disorders, and specification of the process character of addictive behaviors. Neurosci Biobehav Rev.

[34] Brand M, Young KS, Laier C, Wölfling K, Potenza MN (2016). Integrating psychological and neurobiological considerations regarding the development and maintenance of specific Internet-use disorders: An Interaction of Person-Affect-Cognition-Execution (I-PACE) model. Neurosci Biobehav Rev.

[35] Elhai JD, Tiamiyu MF, Weeks JW, Levine JC, Picard KJ, Hall BJ (2018). Depression and emotion regulation predict objective smartphone use measured over one week. Personal Individ Differ.

[36] Boase J, Ling R (2013). Measuring mobile phone use: self-report versus log data. J Comput-Mediat Commun.

[37] Koc M, Gulyagci S (2013). Facebook addiction among turkish college students: the role of psychological health, demographic, and usage characteristics. Cyberpsychology Behav Soc Netw.

[38] Hong FY, Huang DH, Lin HY, Chiu SL (2014). Analysis of the psychological traits, Facebook usage, and Facebook addiction model of Taiwanese university students. Telemat Inform.

[39] Blackwell D, Leaman C, Tramposch R, Osborne C, Liss M (2017). Extraversion, neuroticism, attachment style and fear of missing out as predictors of social media use and addiction. Pers Individ Differ.

[40] Pontes HM, Taylor M, Stavropoulos V (2018). Beyond, “facebook addiction”: The role of cognitive-related factors and psychiatric distress in social networking site addiction. Cyberpsychol Behav Soc Netw.

[41] Montag C, Müller M, Pontes HM, Elhai JD. On fear of missing out, social networks use disorder tendencies and meaning in life. BMC Psychol. accepted; 10.1186/s40359-023-01342-9 PMC10601113 37884983

[42] Montag C, Markett S (2023). Social media use and everyday cognitive failure: investigating the fear of missing out and social networks use disorder relationship. BMC Psychiatry.

[43] Andreassen CS (2015). Online social network site addiction: a comprehensive review. Curr Addict Rep..

[44] Hussain Z, Griffiths MD (2021). The associations between problematic social networking site use and sleep quality, attention-deficit hyperactivity disorder, depression, anxiety and stress. Int J Ment Health Addict.

[45] Hussain Z, Starcevic V (2020). Problematic social networking site use: a brief review of recent research methods and the way forward. Curr Opin Psychol.

[46] Mok WT, Sing R, Jiang X, See SL. Investigation of social media on depression. In: The 9th International Symposium on Chinese Spoken Language Processing. 2014. 488–91.

[47] Kuss DJ, Griffiths MD (2011). Online social networking and addiction—a review of the psychological literature. Int J Environ Res Public Health..

[48] Ponnusamy S, Iranmanesh M, Foroughi B, Hyun SS (2020). Drivers and outcomes of Instagram addiction: psychological well-being as moderator. Comput Hum Behav.

[49] Zivnuska S, Carlson JR, Carlson DS, Harris RB, Harris KJ (2019). Social media addiction and social media reactions: the implications for job performance. J Soc Psychol.

[50] Marino C, Canale N, Melodia F, Spada MM, Vieno A (2021). The overlap between problematic smartphone use and problematic social media use: a systematic review. Curr Addict Rep.

[51] Montag C, Sindermann C, Rozgonjuk D, Yang S, Elhai JD, Yang H (2021). Investigating links between fear of COVID-19, neuroticism, social networks use disorder, and smartphone use disorder tendencies. Front Psychol.

[52] Montag C, Wegmann E, Sariyska R, Demetrovics Z, Brand M (2021). How to overcome taxonomical problems in the study of Internet use disorders and what to do with “smartphone addiction”?. J Behav Addict.

[53] Sha P, Sariyska R, Riedl R, Lachmann B, Montag C (2019). Linking internet communication and smartphone use disorder by taking a closer look at the facebook and whatsapp applications. Addict Behav Rep.

[54] Rozgonjuk D, Sindermann C, Elhai JD, Christensen AP, Montag C (2020). Associations between symptoms of problematic smartphone, Facebook, WhatsApp, and Instagram use: an item-level exploratory graph analysis perspective. J Behav Addict.

[55] Radtke T, Apel T, Schenkel K, Keller J, von Lindern E (2022). Digital detox: An effective solution in the smartphone era? A systematic literature review. Mob Media Commun.

[56] Fernandez DP, Kuss DJ, Griffiths MD (2020). Short-term abstinence effects across potential behavioral addictions: a systematic review. Clin Psychol Rev.

[57] Brown L, Kuss DJ (2020). Fear of missing out, mental wellbeing, and social connectedness: a seven-day social media abstinence trial. Int J Environ Res Public Health.

[58] Lambert J, Barnstable G, Minter E, Cooper J, McEwan D (2022). Taking a one-week break from social media improves well-being, depression, and anxiety: a randomized controlled trial. Cyberpsychol Behav Soc Netw.

[59] Vally Z, D’Souza CG (2019). Abstinence from social media use, subjective well-being, stress, and loneliness. Perspect Psychiatr Care.

[60] Brailovskaia J, Delveaux J, John J, Wicker V, Noveski A, Kim S, et al. Finding the “sweet spot” of smartphone use: Reduction or abstinence to increase well-being and healthy lifestyle?! An experimental intervention study. J Exp Psychol Appl. 2022. Cited 2022 Jun 1; Available from: https://psycnet.apa.org/fulltext/2022-50916-001.pdf. 10.1037/xap0000430 35389685

[61] Brailovskaia J, Meier-Faust J, Schillack H, Margraf J (2022). A two-week gaming abstinence reduces Internet Gaming Disorder and improves mental health: an experimental longitudinal intervention study. Comput Hum Behav.

[62] Huang C (2017). Time spent on social network sites and psychological well-being: a meta-analysis. Cyberpsychol Behav Soc Netw.

[63] Perloff RM (2014). Social media effects on young women’s body image concerns: theoretical perspectives and an agenda for research. Sex Roles..

[64] Rozgonjuk D, Ignell J, Mech F, Rothermund E, Gündel H, Montag C (2023). Smartphone and Instagram use, body dissatisfaction, and eating disorders: investigating the associations using self-report and tracked data. J Eat Disord.

[65] Andsager JL (2014). Research directions in social media and body image. Sex Roles..

[66] Burnette CB, Kwitowski MA, Mazzeo SE (2017). “I don’t need people to tell me I’m pretty on social media:” a qualitative study of social media and body image in early adolescent girls. Body Image..

[67] Kannen C. SurveyCoder. 2020. Cited 2021 May 16. Available from: https://www.surveycoder.com/.

[68] Calogero RM, Thompson KJ, Chrisler JC, McCreary DR (2010). Gender and body image. Handbook of gender research in psychology.

[69] Stevens MW, Dorstyn D, Delfabbro PH, King DL (2021). Global prevalence of gaming disorder: a systematic review and meta-analysis. Aust N Z J Psychiatry.

[70] Wegmann E, Oberst U, Stodt B, Brand M (2017). Online-specific fear of missing out and Internet-use expectancies contribute to symptoms of Internet-communication disorder. Addict Behav Rep.

[71] Riordan BC, Cody L, Flett JAM, Conner TS, Hunter J, Scarf D (2020). The development of a single item FoMO (Fear of Missing Out) scale. Curr Psychol.

[72] Kwon M, Kim DJ, Cho H, Yang S (2013). The Smartphone Addiction Scale: Development And Validation Of A Short Version For Adolescents. PLOS One.

[73] Haug S, Castro RP, Kwon M, Filler A, Kowatsch T, Schaub MP (2015). Smartphone use and smartphone addiction among young people in Switzerland. J Behav Addict.

[74] Kroenke K, Spitzer RL, Williams JBW, Löwe B (2009). An ultra-brief screening scale for anxiety and depression: The PHQ–4. Psychosomatics..

[75] Hughes ME, Waite LJ, Hawkley LC, Cacioppo JT (2004). A short scale for measuring loneliness in large surveys: results from two population-based studies. Res Aging.

[76] Montag C, Schivinski B, Sariyska R, Kannen C, Demetrovics Z, Pontes HM (2019). Psychopathological symptoms and gaming motives in disordered gaming—a psychometric comparison between the WHO and APA diagnostic frameworks. J Clin Med.

[77] Gardner RM, Jappe LM, Gardner L (2009). Development and validation of a new figural drawing scale for body-image assessment: the BIAS-BD. J Clin Psychol.

[78] Vossbeck-Elsebusch AN, Waldorf M, Legenbauer T, Bauer A, Cordes M, Vocks S (2014). German version of the Multidimensional Body-Self Relations Questionnaire – Appearance Scales (MBSRQ-AS): Confirmatory factor analysis and validation. Body Image.

[79] Ahorsu DK, Lin CY, Imani V, Saffari M, Griffiths MD, Pakpour AH (2020). The fear of COVID-19 scale: development and initial validation. Int J Ment Health Addict.

[80] Fatfouta R, Rogoza R (2021). Psychometric Properties and Factor Structure of the German Version of the Fear of COVID-19 Scale. OMEGA - J Death Dying.

[81] Craig CL, Marshall AL, Sjöström M, Bauman AE, Booth ML, Ainsworth BE, et al. International Physical Activity Questionnaire: 12-Country Reliability and Validity: Med Sci Sports Exerc. 2003;35(8):1381–95. 10.1249/01.MSS.0000078924.61453.FB 12900694

[82] Breyer B, Bluemke M. Deutsche Version der Positive and Negative Affect Schedule PANAS (GESIS Panel). Zusammenstellung Sozialwissenschaftlicher Items Skalen ZIS. 2016. Cited 2022 Jun 1; Available from: http://zis.gesis.org/DoiId/zis242.

[83] Watson D, Clark LA, Tellegen A (1988). Development and validation of brief measures of positive and negative affect: the PANAS scales. J Pers Soc Psychol.

[84] Cohen S, Kamarck T, Mermelstein R (1983). A global measure of perceived stress. J Health Soc Behav.

[85] Schneider EE, Schönfelder S, Domke-Wolf M, Wessa M (2020). Measuring stress in clinical and nonclinical subjects using a German adaptation of the Perceived Stress Scale. Int J Clin Health Psychol..

[86] Diener E, Emmons RA, Larsen RJ, Griffin S (1985). The Satisfaction With Life Scale. J Pers Assess.

[87] Janke S, Glöckner-Rist A. Deutsche Version der Satisfaction with Life Scale (SWLS). Zusammenstellung Sozialwissenschaftlicher Items Skalen ZIS. 2012. Cited 2022 Jul 2; Available from: http://zis.gesis.org/DoiId/zis147.

[88] R Core Team. R: A Language and Environment for Statistical Computing. Vienna, Austria: R Foundation for Statistical Computing; 2022. Available from: https://www.R-project.org/.

[89] Grimm KJ, Ram N, Estabrook R. Growth modeling: structural equation and multilevel modeling approaches. New York, NY: Guilford Press; 2017. 537 p. (Methodology in the social sciences).

[90] Bates D, Mächler M, Bolker B, Walker S. Fitting Linear Mixed-Effects Models Using lme4. J Stat Softw. 2015.;67(1). Cited 2023 Jul 23. Available from: http://www.jstatsoft.org/v67/i01/.

[91] Kuznetsova A, Brockhoff PB, Christensen RHB. lmerTest Package: Tests in Linear Mixed Effects Models. J Stat Softw. 2017;82(13). Cited 2023 Jul 23. Available from: http://www.jstatsoft.org/v82/i13/.

[92] Sullivan BM, George AM. The association of motives with problematic smartphone use: A systematic review. Cyberpsychology J Psychosoc Res Cyberspace. 2023;17(1). Cited 2023 Jul 15. Available from: https://cyberpsychology.eu/article/view/20905.

[93] Zhang KZK, Chen C, Lee MKO. Understanding the role of motives in smartphone addiction. In Proceeding of the 19th Pacific Asia Conference on Information Systems (PACIS 2014) Pacific Asia Conference on Information Systems. https://aisel.aisnet.org/pacis2014/131.

[94] Elhai JD, Levine JC, O’Brien KD, Armour C (2018). Distress tolerance and mindfulness mediate relations between depression and anxiety sensitivity with problematic smartphone use. Comput Hum Behav.

[95] Elhai JD, Yang H, Fang J, Bai X, Hall BJ (2020). Depression and anxiety symptoms are related to problematic smartphone use severity in Chinese young adults: Fear of missing out as a mediator. Addict Behav.

[96] Wang Y, Yang H, Montag C, Elhai JD. Boredom proneness and rumination mediate relationships between depression and anxiety with problematic smartphone use severity. Curr Psychol. 2020; Cited 2021 Mar 9. 10.1007/s12144-020-01052-0.

[97] Elhai JD, Vasquez JK, Lustgarten SD, Levine JC, Hall BJ (2018). Proneness to boredom mediates relationships between problematic smartphone use with depression and anxiety severity. Soc Sci Comput Rev.

[98] Rozgonjuk D, Sindermann C, Elhai JD, Montag C (2021). Individual differences in Fear of Missing Out (FoMO): Age, gender, and the Big Five personality trait domains, facets, and items. Personal Individ Differ.

[99] Añez E, Fornieles-Deu A, Fauquet-Ars J, López-Guimerà G, Puntí-Vidal J, Sánchez-Carracedo D (2018). Body image dissatisfaction, physical activity and screen-time in Spanish adolescents. J Health Psychol.

[100] Hrafnkelsdottir SM, Brychta RJ, Rognvaldsdottir V, Chen KY, Johannsson E, Guðmundsdottir SL (2022). Screen time and body image in Icelandic adolescents: sex-specific cross-sectional and longitudinal associations. Int J Environ Res Public Health.

[101] Tang L, Rifas-Shiman SL, Field AE, Austin SB, Haines J (2022). Self-reported total screen time and viewing modes are associated with body dissatisfaction, disordered eating, and cosmetic surgery intentions among young adults. Nutrients.

[102] Wang HY, Cheng C (2022). The associations between gaming motivation and internet gaming disorder: systematic review and meta-analysis. JMIR Ment Health.

[103] de Hesselle LC, Rozgonjuk D, Sindermann C, Pontes HM, Montag C (2020). The associations between Big Five personality traits, gaming motives, and self-reported time spent gaming. Personal Individ Differ.

[104] Elhai JD, Gallinari EF, Rozgonjuk D, Yang H (2020). Depression, anxiety and fear of missing out as correlates of social, non-social and problematic smartphone use. Addict Behav.

[105] Anastasi A (1948). The nature of psychological “traits”. Psychol Rev.

[106] Katz E, Blumler JG, Gurevitch M (1973). Uses and Gratifications Research. Public Opin Q.

[107] Whiting A, Williams D (2013). Why people use social media: a uses and gratifications approach. Qual Mark Res Int J.

