# Supplementary material for: Effects of a 14-day social media abstinence on mental health and well-being: results from an experimental study
Source: BMC Psychol. 2024 Mar 13;12:141. doi: 10.1186/s40359-024-01611-1 (PMC10936093; doi:10.1186/s40359-024-01611-1)
Supplement: Supplementary file 1 — Supplementary Material 1. [file 40359_2024_1611_MOESM1_ESM.docx]

# Supplementary Material

**Supplementary Material 1** Descriptive Statistics for all groups and variables

|  | Total sample (n = 138) | Control (n = 35) | Social media (n = 51) | Gaming ( n = 21) | Combined (n = 31) |
| --- | --- | --- | --- | --- | --- |
|  | m (s) | m (s) | m (s) | m (s) | m (s) |
| Age | 23.33 (5.53) | 23.17 (6.99) | 24 (4.63) | 22.90 (6.20) | 22.68 (4.62) |
| FCV | 11.88 (4.37) | 11.63 (3.90) | 11.9 (4.73) | 12.33 (4.61) | 11.84 (4.31) |
| Trait FoMO | 3.19 (0.85) | 3.32 (0.95) | 3.15 (0.81) | 3.20 (0.94) | 3.12 (0.72) |
| State FoMO | 2.38 (0.80) | 2.40 (0.77) | 2.33 (0.82) | 2.49 (0.85) | 2.39 (0.79) |
| PSU | 32.55 (8.19) | 31.94 (9.52) | 32.06 (7.42) | 35.00 (7.77) | 32.39 (8.19) |
| Depression/ Anxiety | 4.07 (2.78) | 4.03 (2.83) | 3.67 (3.08) | 4.57 (2.13) | 4.42 (2.58) |
| Perceived Stress | 6.72 (3.11) | 7.03 (2.60) | 6.29 (3.49) | 7.00 (3.39) | 6.87 (2.85) |
| Loneliness | 5.23 (1.81) | 5.20 (1.92) | 4.73 (1.52) | 5.86 (1.90) | 5.68 (1.89) |
| Subjective Well-being | 23.72 (6.52) | 24.34 (5.31) | 23.94 (7.17) | 24.00 (7.09) | 22.48 (6.38) |
| Positive affect | 2.93 (0.67) | 2.9 (0.64) | 2.94 (0.72) | 3.01 (0.55) | 2.88 (0.73) |
| Appearance Evaluation | 22.7 (5.79) | 21.66 (5.21) | 22.92 (6.38) | 23.95 (5.53) | 22.65 (5.60) |
| Appearance Orientation | 37.93 (7.83) | 37.8 (8.31) | 38.92 (8.23) | 37.90 (6.74) | 36.48 (7.38) |
| Body Area Satisfaction | 29.05 (5.90) | 29.2 (4.72) | 28.69 (6.53) | 29.90 (5.97) | 28.9 (6.18) |
| Overweight Preoccupation | 9.11 (3.63) | 9.14 (3.52) | 9.00 (3.42) | 10.33 (4.10) | 8.42 (3.74) |
| Self-classified Weight | 5.99 (1.20) | 5.94 (1.00) | 6.08 (1.25) | 6.19 (1.08) | 5.74 (1.39) |
| Screentime (min) | 248.15 (120.39) | 243.12 (119.88) | 246.18 (121.15) | 276.87 (112.92) | 237.6 (127.31) |
| MET | 5449.57 (7621.03) | 5191.55 (5308.96) | 6385.73 (10957) | 2447.84 (694.68) | 5133.21 (2446.72) |
| BID | 2.44 (4.74) | 2.42 (4.62) | 2.79 (5.18) | 3.49 (4.71) | 1.18 (4.02) |

**Note:** FCV = Fear of COVID-19, MET = total weekly metabolic equivalents, BID = Body image dissatisfaction.

**Supplementary Material 2** Model for daily measured perceived stress

|  | Fixed effects | | | | | | | | | Random effect | Fit | |
| --- | --- | --- | --- | --- | --- | --- | --- | --- | --- | --- | --- | --- |
|  | Intercept | Day | PSU | FCV | BID | Stress baseline | | G1 | Day * G1 | $\sigma_{u\left( Intercept \right)}^{2}$ | AIC | BIC |
| M1 | 1.04 (1.00) | 0.00 (0.02) | -0.02 (0.03) | **0.13* (0.06)** | 0.06 (0.04) | **0.51*** (0.08)** |  | |  | 3.48 | 5280.70 | 5320.94 |
| M2 | 1.36 (1.04) | -0.01 (0.03) | -0.02 (0.03) | **0.14* (0.06)** | 0.06 (0.04) | **0.49*** (0.08)** | | -0.56 (0.49) | 0.02 (0.03) | 3.48 | 5288.13 | 5338.44 |

**Note:** PSU, FCV, BID, and Stress at baseline. G1: 0 = control group, 1 = social media abstinence group. AIC = Akaike Information Criterion, BIC = Bayesian Information Criterion, **p* < .05, ***p* < .01, ****p* < .001.

**Supplementary Material 3** Models for Perceived Stress, Subjective well-being, positive affect and weekly metabolic equivalents for control group and social media abstinence group

|  |  |  | Perceived  Stress | SWLS | Positive  affect | MET |
| --- | --- | --- | --- | --- | --- | --- |
|  |  |  | b (SE) | b (SE) | b (SE) | b (SE) |
| M1 | Fixed effects | Intercept | **5.66^***^** | **23.90^***^** | **3.02^***^** | **5060.44^**^** |
|  |  |  | **(0.33)** | **(0.69)** | **(0.08)** | **(1772.34)** |
|  |  | T1 | **-0.93^***^** | -0.21 | 0.10 | -819.65 |
|  |  |  | **(0.28)** | (0.37) | (0.07) | (2331.59) |
|  |  | T2 | 0.46 | 0.18 | -0.04 | 2844.49 |
|  |  |  | (0.29) | (0.39) | (0.07) | (2571.55) |
|  | Random Effects | $\sigma_{u\left( Intercept \right)}^{2}$ | 7.72 | 35.52 | 0.32 | 2147854.16 |
|  | Fit | AIC | 1194.88 | 1413.26 | 465.29 | 2281.11 |
|  |  | BIC | 1219.50 | 1437.88 | 482.88 | 2294.56 |
| M2 | Fixed  effects | Intercept | **2.51^*^** | **29.91^***^** | **3.57^***^** | **13505.66^**^** |
|  |  |  | **(1.26)** | **(2.82)** | **(0.29)** | **(4884.94)** |
|  |  | T1 | -0.51 | -1.03 | -0.13 | -226.39 |
|  |  |  | (0.43) | (0.58) | (0.10) | (3721.19) |
|  |  | T2 | 0.09 | 0.37 | 0.17 | 161.04 |
|  |  |  | (0.44) | (0.59) | (0.10) | (3971.29) |
|  |  | Group | **-1.52^*^** | 1.14 | **0.44^**^** | 311.24 |
|  |  |  | **(0.62)** | (1.29) | **(0.15)** | (3667.41) |
|  |  | FCV | **0.29^***^** | **-0.61^***^** | **-0.05^***^** | -37.10 |
|  |  |  | **(0.06)** | **(0.14)** | **(0.01)** | (196.24) |
|  |  | PSU | 0.02 | 0.01 | -0.01 | **-300.73^*^** |
|  |  |  | (0.03) | (0.07) | (0.01) | **(134.74)** |
|  |  | T1 * Group | -0.70 | 1.38 | **0.38^**^** | -1917.30 |
|  |  |  | (0.56) | (0.75) | **(0.13)** | (4723.00) |
|  |  | T2 * Group | 0.62 | -0.28 | **-0.34^*^** | 6380.13 |
|  |  |  | (0.59) | (0.78) | **(0.14)** | (5156.24) |
|  |  | BID |  |  |  | 372.90 |
|  |  |  |  |  |  | (210.59) |
|  | Random  effects | $\sigma_{u\left( Intercept \right)}^{2}$ | 5.39 | 30.23 | 0.26 | 0.00 |
|  |  | $\sigma_{u\left( Time \right)}^{2}$ | 0.85 | 0.41 |  |  |
|  | Fit | AIC | 1185.25 | 1402.58 | 469.99 | 2189.68 |
|  |  | BIC | 1227.46 | 1444.78 | 505.16 | 2219.28 |

**Note:** T1 = baseline vs. end, T2 = end vs. follow-up, Group: 0 = control, 1 = social media abstinence, AIC = Akaike Information Criterion, BIC = Bayesian Information Criterion, PSU = Problematic Smartphone use, SWLS = subjective well-being, MET = total weekly metabolic equivalents, FCV = Fear of COVID-19, BID = Body image dissatisfaction, **p* < .05, ***p* < .01, ****p* < .001.

**Supplementary Material 4** Correlation Table with Holm corrected p-values and 95% confidence intervals

|  | 1. Age | 2 | 3 | 4 | 5 | 6 | 7 | 8 | 9 | 10 | 11 | 12 | 13 | 14 | 15 | 16 | 17 |
| --- | --- | --- | --- | --- | --- | --- | --- | --- | --- | --- | --- | --- | --- | --- | --- | --- | --- |
| 1. FCV | .08 |  |  |  |  |  |  |  |  |  |  |  |  |  |  |  |  |
|  | [-.09, .24] |  |  |  |  |  |  |  |  |  |  |  |  |  |  |  |  |
| 1. trait FoMO | -.22 | .19 |  |  |  |  |  |  |  |  |  |  |  |  |  |  |  |
|  | [-.38, -.06] | [.02, .34] |  |  |  |  |  |  |  |  |  |  |  |  |  |  |  |
| 1. state FoMO | -.14 | **.31*** | **.46***** |  |  |  |  |  |  |  |  |  |  |  |  |  |  |
|  | [-.30, .02] | [.15, .45] | [.31, .58] |  |  |  |  |  |  |  |  |  |  |  |  |  |  |
| 1. PSU | -.13 | .27 | **.31*** | **.68***** |  |  |  |  |  |  |  |  |  |  |  |  |  |
|  | [-.29, .04] | [.11, .42] | [.16, .46] | [.57, .76] |  |  |  |  |  |  |  |  |  |  |  |  |  |
| 1. Depression/ Anxiety | -.08 | **.32*** | .16 | .24 | **.30*** |  |  |  |  |  |  |  |  |  |  |  |  |
|  | [-.25, .08] | [.16, .46] | [-.01, .31] | [.07, .39] | [.14, .44] |  |  |  |  |  |  |  |  |  |  |  |  |
| 1. Stress | .04 | **.36**** | .16 | .24 | .26 | **.65***** |  |  |  |  |  |  |  |  |  |  |  |
|  | [-.13, .20] | [.20, .50] | [-.01, .31] | [.07, .39] | [.10, .41] | [.54, .74] |  |  |  |  |  |  |  |  |  |  |  |
| 1. Loneliness | 0 | .23 | **.39***** | .24 | .33* | **.45***** | **.36**** |  |  |  |  |  |  |  |  |  |  |
|  | [-.17, .17] | [.07, .38] | [.24, .53] | [.07, .39] | [.17, .47] | [.31, .57] | [.21, .50] |  |  |  |  |  |  |  |  |  |  |
| 1. SWLS | -.24 | **-.30*** | -.10 | -.08 | -.09 | **-.47***** | **-.50***** | **-.42***** |  |  |  |  |  |  |  |  |  |
|  | [-.40, -.08] | [-.44, -.14] | [-.26, .07] | [-.24, .09] | [-.25, .08] | [-.59, -.33] | [-.61, -.36] | [-.55, -.28] |  |  |  |  |  |  |  |  |  |
| 1. positive affect | -.18 | -.26 | -.23 | -.07 | -.10 | **-.40***** | **-.48***** | **-.38***** | **.49***** |  |  |  |  |  |  |  |  |
|  | [-.34, -.01] | [-.41, -.10] | [-.38, -.07] | [-.23, .10] | [-.26, .07] | [-.53, -.25] | [-.60, -.34] | [-.51, -.22] | [.35, .60] |  |  |  |  |  |  |  |  |
| 1. MET | .17 | -.16 | -.15 | -.15 | -.26 | -.08 | -.07 | -.06 | .13 | .12 |  |  |  |  |  |  |  |
|  | [-.08, .40] | [-.39, .09] | [-.38, .10] | [-.38, .10] | [-.48, -.02] | [-.32, .17] | [-.31, .18] | [-.30, .19] | [-.12, .37] | [-.13, .36] |  |  |  |  |  |  |  |
| 1. screen- time | -.14 | .21 | .11 | .14 | .14 | **.30*** | .23 | .19 | -.21 | -.22 | -.19 |  |  |  |  |  |  |
|  | [-.30, .03] | [.05, .37] | [-.06, .27] | [-.03, .30] | [-.03, .30] | [.14, .44] | [.06, .38] | [.03, .35] | [-.37, -.05] | [-.38, -.06] | [-.42, .06] |  |  |  |  |  |  |
| 1. BID | .28 | -.08 | -.06 | .01 | .04 | .13 | .15 | -.01 | -.19 | -.12 | -.04 | -.02 |  |  |  |  |  |
|  | [.12, .42] | [-.24, .09] | [-.23, .10] | [-.16, .17] | [-.13, .21] | [-.04, .29] | [-.01, .31] | [-.18, .16] | [-.35, -.02] | [-.28, .05] | [-.29, .20] | [-.19, .15] |  |  |  |  |  |
| 1. Appearance  Evaluation | -.20 | -.25 | -.18 | -.17 | -.17 | **-.37**** | **-.47***** | **-.40***** | **.59***** | **.45***** | .08 | -.09 | **-.36**** |  |  |  |  |
|  | [-.36, -.04] | [-.40, -.09] | [-.34, -.01] | [-.33, -.00] | [-.33, -.01] | [-.50, -.21] | [-.59, -.33] | [-.53, -.25] | [.47, .69] | [.31, .58] | [-.17, .32] | [-.26, .07] | [-.50, -.20] |  |  |  |  |

**Supplementary Material 4** (continued)

|  | 1. Age | 2 | 3 | 4 | 5 | 6 | 7 | 8 | 9 | 10 | 11 | 12 | 13 | 14 | 15 | 16 | 17 |
| --- | --- | --- | --- | --- | --- | --- | --- | --- | --- | --- | --- | --- | --- | --- | --- | --- | --- |
| 1. Appearance Orientation | -.15 | .06 | .26 | **.30*** | .28 | -.02 | .05 | -.04 | -.01 | -.06 | -.30 | .08 | .01 | .05 |  |  |  |
|  | [-.31, .02] | [-.11, .22] | [.10, .41] | [.14, .45] | [.11, .42] | [-.19, .15] | [-.12, .21] | [-.21, .13] | [-.17, .16] | [-.23, .10] | [-.51, -.05] | [-.09, .24] | [-.15, .18] | [-.12, .22] |  |  |  |
| 1. Body Area  Satisfaction | -.19 | -.24 | -.15 | -.21 | .17 | **-.30*** | **-.46***** | **-.34**** | **.55***** | **.43***** | .13 | -.13 | **-.39***** | **.84***** | -.04 |  |  |
|  | [-.35, -.03] | [-.39, -.08] | [-.31, .02] | [-.37, -.05] | [-.33, -.00] | [-.45, -.14] | [-.58, -.32] | [-.48, -.18] | [.42, .65] | [.29, .56] | [-.12, .36] | [-.29, .04] | [-.52, -.24] | [.78, .88] | [-.20, .13] |  |  |
| 1. Overweight Preoccupation | -.01 | .08 | .13 | .14 | .12 | .12 | .17 | .13 | -.18 | -.13 | -.21 | -.06 | **.47***** | **-.30*** | **.33**** | **-.36**** |  |
|  | [-.18, .16] | [-.09, .24] | [-.04, .29] | [-.03, .30] | [-.05, .28] | [-.05, .28] | [.00, .33] | [-.04, .29] | [-.34, -.02] | [-.29, .04] | [-.43, .04] | [-.22, .11] | [.33, .59] | [-.45, -.14] | [.18, .47] | [-.50, -.20] |  |
| 1. Self-classified  weight | .27 | -.08 | -.05 | .03 | .14 | .11 | .08 | .04 | -.10 | -.09 | -.06 | -.04 | **.75***** | -.27 | .05 | -.23 | **.38***** |
|  | [.11, .42] | [-.25, .08] | [-.21, .12] | [-.14, .20] | [-.02, .30] | [-.06, .27] | [-.09, .24] | [-.13, .20] | [-.27, .06] | [-.26, .07] | [-.30, .19] | [-.21, .13] | [.66, .81] | [-.42, -.11] | [-.12, .21] | [-.38, -.07] | [.23, .51] |

**Note:** FCV = Fear of COVID-19, FoMO = Fear of Missing Out, PSU = Problematic smartphone use, SWLS = subjective well-being, BID = Body Image Dissatisfaction, MET = total weekly metabolic equivalents. Holm corrected *p*-values: **p* < .05, ***p* < .01, ****p* < .001. 95% Confidence Intervals.

**Supplementary Material 5** Multilevel Models for all variables, including all groups

|  |  |  | Depression/ Anxiety | Screentime | Perceived  stress | PSU | Positive  affect | Loneliness | SWLS | State  FoMO | Trait  FoMO |
| --- | --- | --- | --- | --- | --- | --- | --- | --- | --- | --- | --- |
|  |  |  | b (SE) | b (SE) | b (SE) | b (SE) | b (SE) | b (SE) | b (SE) | b (SE) | b (SE) |
| M1 | Fixed effects | Intercept | 3.46^***^ | 229.58^***^ | 6.04^***^ | 29.28^***^ | 2.97^***^ | 4.36^***^ | 23.86^***^ | 2.12^***^ | 2.73^***^ |
|  |  |  | (0.23) | (10.57) | (0.27) | (0.68) | (0.06) | (0.14) | (0.56) | (0.06) | (0.07) |
|  |  | T1 | **-0.61^**^** | **-18.57^*^** | **-0.68^**^** | **-3.27^***^** | 0.04 | **-0.88^***^** | 0.14 | **-0.26^***^** | **-0.46^***^** |
|  |  |  | (0.19) | (9.04) | (0.23) | (0.56) | (0.05) | (0.12) | (0.27) | (0.05) | (0.05) |
|  |  | T2 | -0.15 | 5.13 | 0.24 | **-1.50^*^** | 0.01 | 0.06 | 0.24 | -0.02 | **-0.13^*^** |
|  |  |  | (0.20) | (9.39) | (0.24) | (0.58) | (0.05) | (0.13) | (0.28) | (0.05) | (0.06) |
|  | Fit | AIC | 1769.69 | 4825.89 | 1933.75 | 2661.64 | 710.22 | 1438.00 | 2247.28 | 708.05 | 843.29 |
|  |  | BIC | 1789.65 | 4845.85 | 1961.69 | 2689.58 | 730.18 | 1465.94 | 2275.22 | 735.99 | 871.23 |
|  | Random effects | $\sigma_{u\left( Intercept \right)}^{2}$ | 4.96 | 9780.73 | 6.53 | 48.48 | 0.30 | 1.97 | 37.89 | 0.47 | 0.56 |
|  |  | $\sigma_{u\left( Time \right)}^{2}$ |  |  | 0.57 | 5.33 |  | 0.20 | 1.19 | 0.07 | 0.08 |
| M2 | Fixed effects | Intercept | -0.16 | 142.83^**^ | 2.46^*^ | 23.25^***^ | 3.29^***^ | 2.35^***^ | 28.44^***^ | 0.47^*^ | 1.93^***^ |
|  |  |  | (0.92) | (44.09) | (1.07) | (2.01) | (0.24) | (0.56) | (2.47) | (0.22) | (0.30) |
|  |  | T1 | -0.51 | 1.14 | -0.51 | **-2.91^**^** | -0.13 | **-0.80^**^** | -1.03 | **-0.18^*^** | **-0.41^***^** |
|  |  |  | (0.38) | (17.95) | (0.46) | (1.11) | (0.10) | (0.25) | (0.54) | (0.09) | (0.11) |
|  |  | T2 | -0.08 | -6.09 | 0.09 | -0.33 | 0.16 | 0.19 | 0.32 | -0.10 | -0.19 |
|  |  |  | (0.39) | (18.32) | (0.47) | (1.13) | (0.10) | (0.25) | (0.55) | (0.09) | (0.11) |
|  |  | G1 | -0.57 | -36.95 | **-1.50^*^** | -1.89 | **0.43^**^** | -0.27 | 1.10 | -0.15 | **-0.37^*^** |
|  |  |  | (0.56) | (26.92) | (0.66) | (1.68) | (0.15) | (0.34) | (1.39) | (0.13) | (0.18) |
|  |  | G2 | 0.25 | 27.43 | -0.11 | 3.10 | 0.21 | 0.06 | 1.61 | -0.21 | -0.18 |
|  |  |  | (0.71) | (34.02) | (0.83) | (2.11) | (0.19) | (0.44) | (1.76) | (0.17) | (0.22) |
|  |  | G3 | 0.17 | -32.21 | 0.03 | 1.58 | 0.11 | 0.02 | 0.04 | -0.18 | -0.14 |
|  |  |  | (0.63) | (30.25) | (0.74) | (1.88) | (0.17) | (0.39) | (1.57) | (0.15) | (0.20) |
|  |  | FCV | **0.18^***^** | 3.18 | **0.20^***^** | **0.50^***^** | **-0.04^**^** | **0.10^***^** | **-0.44^***^** | **0.02^*^** | **0.03^*^** |
|  |  |  | (0.05) | (2.16) | (0.05) | (0.13) | (0.01) | (0.03) | (0.12) | (0.01) | (0.01) |
|  |  | PSU | **0.05^*^** | 2.02 | 0.05 |  | -0.00 | 0.03 | 0.00 | **0.05^***^** | **0.02^*^** |
|  |  |  | (0.02) | (1.17) | (0.03) |  | (0.01) | (0.01) | (0.07) | (0.01) | (0.01) |
|  |  | T1 * G1 | -0.15 | -38.90 | -0.70 | -1.87 | **0.38^**^** | 0.23 | **1.38^*^** | -0.07 | -0.19 |
|  |  |  | (0.49) | (23.31) | (0.59) | (1.44) | (0.13) | (0.32) | (0.70) | (0.12) | (0.14) |
|  |  | T2 * G1 | 0.07 | 25.34 | 0.62 | -2.04 | **-0.34^*^** | -0.05 | -0.29 | 0.04 | 0.18 |
|  |  |  | (0.51) | (24.11) | (0.62) | (1.49) | (0.13) | (0.33) | (0.73) | (0.12) | (0.15) |
|  |  |  |  |  |  |  |  |  |  |  |  |

**Supplementary Material 5** (continued)

|  |  |  | Depression/ Anxiety | Screentime | Perceived  stress | PSU | Positive  affect | Loneliness | SWLS | State  FoMO | Trait  FoMO |
| --- | --- | --- | --- | --- | --- | --- | --- | --- | --- | --- | --- |
|  |  |  | b (SE) | b (SE) | b (SE) | b (SE) | b (SE) | b (SE) | b (SE) | b (SE) | b (SE) |
|  |  | T1* G2 | -0.01 | 2.09 | 0.23 | 0.39 | 0.06 | -0.44 | 1.65 | -0.15 | 0.02 |
|  |  |  | (0.62) | (29.31) | (0.74) | (1.81) | (0.16) | (0.40) | (0.87) | (0.15) | (0.18) |
|  |  | T2 * G2 | -0.49 | -28.05 | -0.89 | -0.75 | -0.11 | -0.30 | 0.48 | 0.23 | -0.08 |
|  |  |  | (0.64) | (30.57) | (0.78) | (1.90) | (0.17) | (0.42) | (0.92) | (0.16) | (0.19) |
|  |  | T1 * G3 | -0.16 | -25.12 | 0.26 | 1.24 | 0.11 | -0.43 | **1.80^*^** | -0.15 | 0.08 |
|  |  |  | (0.55) | (26.19) | (0.66) | (1.61) | (0.15) | (0.36) | (0.78) | (0.13) | (0.16) |
|  |  | T2 * G3 | -0.15 | 25.44 | 0.19 | -1.57 | -0.04 | -0.31 | -0.14 | 0.16 | -0.01 |
|  |  |  | (0.56) | (26.78) | (0.68) | (1.66) | (0.15) | (0.37) | (0.80) | (0.14) | (0.16) |
|  | Fit | AIC | 1769.93 | 4753.95 | 1925.49 | 2630.77 | 742.53 | 1442.51 | 2238.39 | 706.69 | 875.62 |
|  |  | BIC | 1833.79 | 4817.82 | 1997.34 | 2698.63 | 806.39 | 1514.36 | 2310.23 | 778.54 | 947.47 |
|  | Random Effects | $\sigma_{u\left( Intercept \right)}^{2}$ | 4.07 | 9396.22 | 5.14 | 43.61 | 0.28 | 1.54 | 35.22 | 0.23 | 0.50 |
|  |  | $\sigma_{u\left( Time \right)}^{2}$ |  |  | 0.62 | 4.75 |  | 0.17 | 1.12 | 0.07 | 0.09 |

**Note:** T1 = baseline vs. end, T2 = end vs. follow-up, G1: 0 = control, 1 = social media abstinence, G2: 0 = control, 1 = gaming abstinence, G3: 0 = control, 1 = combined gaming and social media abstinence, AIC = Akaike Information Criterion, BIC = Bayesian Information Criterion, PSU = Problematic Smartphone use, SWLS = subjective well-being, FCV = Fear of COVID-19. **p* < .05, ***p* < .01, ****p* < .001.

**Supplementary Material 5** (continued, body image associated models)

|  |  |  | BID | MET | Appearance Evaluation | Appearance Orientation | Overweight Preoccupation | Body-area Satisfaction | Self-classified weight |
| --- | --- | --- | --- | --- | --- | --- | --- | --- | --- |
|  |  |  | b (SE) | b (SE) | b (SE) | b (SE) | b (SE) | b (SE) | b (SE) |
| M1 | Fixed effects | Intercept | 1.99^***^ | 4672.16^***^ | 23.62^***^ | 36.96^***^ | 8.89^***^ | 29.52^***^ | 5.88^***^ |
|  |  |  | (0.39) | (1218.77) | (0.52) | (0.67) | (0.32) | (0.50) | (0.10) |
|  |  | T1 | **-0.45^*^** | -754.54 | **0.93^***^** | **-0.98^**^** | -0.22 | 0.47 | **-0.11^*^** |
|  |  |  | (0.19) | (1598.80) | (0.27) | (0.35) | (0.16) | (0.28) | (0.05) |
|  |  | T2 | -0.01 | 1662.55 | -0.12 | **1.10^**^** | -0.09 | 0.13 | 0.02 |
|  |  |  | (0.20) | (1704.25) | (0.28) | (0.36) | (0.17) | (0.29) | (0.05) |
|  | Fit | AIC | 1950.09 | 3375.69 | 2202.09 | 2416.61 | 1827.86 | 2218.51 | 878.54 |
|  |  | BIC | 1978.03 | 3391.16 | 2222.05 | 2444.55 | 1855.80 | 2246.45 | 898.50 |
|  | Random effects | $\sigma_{u\left( Intercept \right)}^{2}$ | 20.08 | 3726544.76 | 31.86 | 54.14 | 11.38 | 27.82 | 1.21 |
|  |  | $\sigma_{u\left( Time \right)}^{2}$ | 0.27 |  |  | 1.32 | 0.41 | 0.94 |  |
| M2 | Fixed effects | Intercept | 2.80 | 10616.49^**^ | 28.43^***^ | 28.77^***^ | 5.52^***^ | 35.53^***^ | 5.09^***^ |
|  |  |  | (1.79) | (3515.08) | (2.11) | (2.99) | (1.25) | (2.04) | (0.32) |
|  |  | T1 | 0.12 | -94.63 | 1.37^*^ | -1.17 | -0.66^*^ | 0.34 | -0.09 |
|  |  |  | (0.37) | (3132.09) | (0.54) | (0.69) | (0.33) | (0.55) | (0.10) |
|  |  | T2 | -0.05 | -255.71 | -0.16 | 0.71 | 0.19 | 0.47 | 0.04 |
|  |  |  | (0.38) | (3341.33) | (0.55) | (0.70) | (0.34) | (0.56) | (0.10) |
|  |  | G1 | -0.55 | 16.17 | 0.91 | 1.89 | 0.30 | 0.36 | 0.04 |
|  |  |  | (1.01) | (3069.06) | (1.20) | (1.68) | (0.72) | (1.19) | (0.19) |
|  |  | G2 | -0.34 | -2560.77 | 2.31 | -1.86 | 1.15 | 0.60 | -0.13 |
|  |  |  | (1.28) | (4605.96) | (1.52) | (2.13) | (0.91) | (1.50) | (0.24) |
|  |  | G3 | -1.26 | -243.79 | 0.04 | -1.06 | 0.30 | -0.59 | 0.06 |
|  |  |  | (1.13) | (3544.59) | (1.35) | (1.90) | (0.81) | (1.34) | (0.21) |
|  |  | FCV | -0.10 | 6.45 | **-0.36^***^** | -0.01 | 0.10 | **-0.33^**^** | -0.01 |
|  |  |  | (0.09) | (147.66) | (0.11) | (0.15) | (0.06) | (0.10) | (0.02) |
|  |  | PSU | 0.03 | **-210.32^*^** | 0.00 | **0.25^**^** | 0.03 | -0.03 | 0.01 |
|  |  |  | (0.05) | (91.29) | (0.06) | (0.08) | (0.03) | (0.05) | (0.01) |
|  |  | T1 * G1 | **-0.95^*^** | -1876.93 | -0.65 | 0.80 | 0.62 | 0.60 | -0.03 |
|  |  |  | (0.48) | (3977.70) | (0.70) | (0.89) | (0.43) | (0.71) | (0.13) |
|  |  | T2 * G1 | 0.09 | 6742.79 | 0.10 | 0.26 | -0.27 | -0.53 | -0.08 |
|  |  |  | (0.50) | (4343.74) | (0.73) | (0.93) | (0.44) | (0.74) | (0.14) |
|  |  | T1* G2 | **-1.40^*^** | 570.67 | -0.80 | -1.21 | 0.51 | -0.96 | -0.15 |
|  |  |  | (0.61) | (6212.35) | (0.88) | (1.12) | (0.53) | (0.89) | (0.16) |
|  |  | T2 * G2 | 0.26 | 988.81 | 1.16 | 1.16 | -0.43 | 0.58 | -0.01 |
|  |  |  | (0.64) | (5965.79) | (0.92) | (1.18) | (0.56) | (0.94) | (0.17) |
|  |  | T1 * G3 | -0.03 | -872.30 | -0.37 | 0.36 | 0.59 | 0.24 | 0.05 |
|  |  |  | (0.54) | (4626.80) | (0.79) | (1.00) | (0.48) | (0.80) | (0.15) |
|  |  | T2 * G3 | -0.17 | 938.62 | -0.73 | 0.58 | -0.53 | -1.02 | 0.02 |
|  |  |  | (0.56) | (4852.11) | (0.81) | (1.03) | (0.49) | (0.82) | (0.15) |
|  |  | BID |  | 292.82 | **-0.53^***^** | 0.00 | **0.37^***^** | **-0.49^***^** | **0.17^***^** |
|  |  |  |  | (149.69) | (0.09) | (0.13) | (0.06) | (0.09) | (0.01) |
|  | Fit | AIC | 1959.54 | 3185.73 | 2178.83 | 2412.67 | 1811.82 | 2197.62 | 832.33 |
|  |  | BIC | 2031.39 | 3238.32 | 2246.69 | 2488.51 | 1887.66 | 2273.46 | 900.18 |
|  | Random effects | $\sigma_{u\left( Intercept \right)}^{2}$ | 20.18 | 0.00 | 24.82 | 50.55 | 8.43 | 21.35 | 0.55 |
|  |  | $\sigma_{u\left( Time \right)}^{2}$ | 0.28 |  |  | 1.40 | 0.42 | 1.01 |  |

**Note:** T1 = baseline vs. end, T2 = end vs. follow-up, G1: 0 = control, 1 = social media abstinence, G2: 0 = control, 1 = gaming abstinence, G3: 0 = control, 1 = combined gaming and social media abstinence, AIC = Akaike Information Criterion, BIC = Bayesian Information Criterion, BID = Body Image dissatisfaction, MET = total weekly metabolic equivalents, FCV = Fear of COVID-19, **p* < .05, ***p* < .01, ****p* < .001.

**Supplementary Material 6** Daily measured depression and anxiety

|  | Fixed effects | | | | | | | | | | | | Random effects | Fit | |
| --- | --- | --- | --- | --- | --- | --- | --- | --- | --- | --- | --- | --- | --- | --- | --- |
|  | Intercept | Day | PSU | FCV | BID | Depression/ Anxiety | G1 | G2 | G3 | Day*G1 | Day*G2 | Day*G3 | $\sigma_{u\left( Intercept \right)}^{2}$ | AIC | BIC |
| M1 | -0.52 (0.58) | 0.01 (0.01) | -0.01 (0.02) | **0.12*** (0.03)** | 0.02 (0.03) | **0.40*** (0.05)** |  |  |  |  |  |  | 1.98 | 7607.90 | 7651.97 |
| M2 | -0.38 (0.62) | -0.04 (0.02) | -0.00 (0.02) | **0.12*** (0.03)** | 0.02 (0.03) | **0.41*** (0.05)** | -0.15 (0.37) | -0.61 (0.47) | -0.31 (0.42) | **0.06* (0.03)** | 0.05 (0.03) | **0.07* (0.03)** | 1.99 | 7627.83 | 7704.95 |

**Note:** G1: 0 = control, 1 = social media abstinence, G2: 0 = control, 1 = gaming abstinence, G3: 0 = control, 1 = combined gaming and social media abstinence, AIC = Akaike Information Criterion, BIC = Bayesian Information Criterion, PSU = Problematic Smartphone use, FCV = Fear of COVID-19, BID = body image dissatisfaction. **p* < .05, ***p* < .01, ****p* < .001.

**Supplementary Material 7** Daily measured perceived stress (PSS) for all groups

|  | Fixed effects | | | | | | | | | | | | Random effects | Fit | |
| --- | --- | --- | --- | --- | --- | --- | --- | --- | --- | --- | --- | --- | --- | --- | --- |
|  | Intercept | Day | PSU | FCV | BID | PSS | G1 | G2 | G3 | Day*G1 | Day*G2 | Day*G3 | $\sigma_{u\left( Intercept \right)}^{2}$ | AIC | BIC |
| M1 | 0.63 (0.79) | 0.01 (0.01) | 0.00 (0.02) | **0.12** (0.04)** | 0.02 (0.04) | **0.50*** (0.06)** |  |  |  |  |  |  | 3.71 | 8493.92 | 8537.99 |
| M2 | 0.87 (0.85) | -0.01 (0.03) | 0.00 (0.02) | **0.12** (0.04)** | 0.03 (0.04) | **0.49*** (0.06)** | -0.55 (0.50) | -0.12 (0.63) | 0.05 (0.56) | 0.02 (0.03) | 0.01 (0.04) | 0.06 (0.04) | 3.68 | 8513.43 | 8590.55 |

**Note:** G1: 0 = control, 1 = social media abstinence, G2: 0 = control, 1 = gaming abstinence, G3: 0 = control, 1 = combined gaming and social media abstinence, AIC = Akaike Information Criterion, BIC = Bayesian Information Criterion, PSU = Problematic Smartphone use, FCV = Fear of COVID-19, BID = body image dissatisfaction. **p* < .05, ***p* < .01, ****p* < .001.

**Supplementary Material 8** Daily measured screentime

|  | Fixed effects | | | | | | | | | | | | | | | | | | | | | | | | | | | | | | | | Random  effects | | | |
| --- | --- | --- | --- | --- | --- | --- | --- | --- | --- | --- | --- | --- | --- | --- | --- | --- | --- | --- | --- | --- | --- | --- | --- | --- | --- | --- | --- | --- | --- | --- | --- | --- | --- | --- | --- | --- |
|  | Intercept | | Day | | Day² | | PSU | FCV | | BID | | Screen time | | G1 | | G2 | | G3 | | Day * G1 | | Day * G2 | | Day * G3 | | Day² * G1 | | Day² * G2 | | | Day² * G3 | | $\sigma_{u\left( Intercept \right)}^{2}$ | | |  |
| M1 | 18.46 (25.12) | | 0.59  (0.48) | |  | | **1.52* (0.68)** | -0.97 (1.29) | | **-2.76* (1.13)** | | **0.72*** (0.05)** | |  | |  | |  | |  | |  | |  | |  | |  | | |  | | 3329.83 | | |  |
| M2 | 14.29 (25.37) | | 2.71 (1.82) | | -0.16 (0.13) | | **1.52* (0.68)** | -0.97 (1.29) | | **-2.77* (1.13)** | | **0.72*** (0.05)** | |  | |  | |  | |  | |  | |  | |  | |  | | |  | | 3334.34 | | |  |
| M3 | 28.09 (27.21) | | 0.15 (0.98) | |  | | **1.51* (0.69)** | -0.97 (1.30) | | **-2.93* (1.15)** | | **0.72*** (0.05)** | | -8.55 (16.04) | | -3.98 (20.36) | | -21.48 (18.08) | | 0.59 (1.26) | | 0.35 (1.58) | | 0.69 (1.41) | |  | |  | | |  | | 3369.53 | | |  |
| M4 | 30.88 (28.05) | | -1.31 (3.66) | | 0.11 (0.27) | | **1.51* (0.69)** | -0.96 (1.30) | | **-2.93* (1.15)** | | **0.72*** (0.05)** | | -24.27 (18.33) | | -9.06 (23.19) | | -23.33 (20.60) | | 8.51 (4.71) | | 2.96 (5.90) | | 1.66 (5.30) | | -0.61 (0.35) | | -0.20 (0.44) | | | -0.08 (0.40) | | 3373.74 | | |  |
|  |  | |  | |  | |  |  | |  | |  | |  | |  | |  | |  | |  | |  | |  | |  | | |  | |  | | |  |
|  | | Model Fit | | | | | | | | | | |  | |  | |  | |  | |  | |  | |  | |  | |  |  | |  | |  |  |  |
|  | |  | | M1 | | M2 | | | M3 | | M4 | |  | |  | |  | |  | |  | |  | |  | |  | |  |  | |  | |  |  |  |
|  | | AIC | | 21589.73 | | 21592.42 | | | 21571.79 | | 21577.35 | |  | |  | |  | |  | |  | |  | |  | |  | |  |  | |  | |  |  |  |
|  | | BIC | | 21633.80 | | 21642.00 | | | 21648.91 | | 21676.50 | |  | |  | |  | |  | |  | |  | |  | |  | |  |  | |  | |  |  |  |

**Note:** G1: 0 = control, 1 = social media abstinence, G2: 0 = control, 1 = gaming abstinence, G3: 0 = control, 1 = combined gaming and social media abstinence, AIC = Akaike Information Criterion, BIC = Bayesian Information Criterion, PSU = Problematic Smartphone use, FCV = Fear of COVID-19, BID = body image dissatisfaction. **p* < .05, ***p* < .01, ****p* < .001.

**Supplementary Material 9** Daily measured loneliness

|  | Fixed effects | | | | | | | | | | | | | | | | | | | | Random effects |
| --- | --- | --- | --- | --- | --- | --- | --- | --- | --- | --- | --- | --- | --- | --- | --- | --- | --- | --- | --- | --- | --- |
|  | Inter- cept | Day | Day² | Day³ | PSU | FCV | BID | Loneliness | G1 | G2 | G3 | Day* G1 | Day* G2 | Day* G3 | Day²* G1 | Day²* G2 | Day²* G3 | Day³* G1 | Day³* G2 | Day³* G3 | $\sigma_{u\left( I \right)}^{2}$ |
| M1 | **1.68*** (0.40)** | 0.00  (0.01) |  |  | -0.00 (0.01) | **0.08*** (0.02)** | **0.04* (0.02)** | **0.29*** (0.05)** |  |  |  |  |  |  |  |  |  |  |  |  | 0.90 |
| M2 | **1.72*** (0.41)** | -0.02  (0.02) | 0.00  (0.00) |  | -0.00 (0.01) | **0.08*** (0.02)** | **0.04* (0.02)** | **0.29*** (0.05)** |  |  |  |  |  |  |  |  |  |  |  |  | 0.90 |
| M3 | **1.89*****  **(0.41)** | **-0.21*** (0.05)** | **0.04*** (0.01)** | **-0.01*** (0.01)** | -0.00 (0.01) | **0.08*** (0.02)** | **0.04* (0.02)** | **0.29*** (0.05)** |  |  |  |  |  |  |  |  |  |  |  |  | 0.91 |
| M4 | **1.54*** (0.43)** | -0.01  (0.01) |  |  | -0.00 (0.01) | **0.08*** (0.02)** | **0.05* (0.02)** | **0.30*** (0.05)** | 0.26 (0.24) | -0.22 (0.31) | 0.05 (0.27) | 0.01  (0.02) | 0.03 (0.02) | **0.04* (0.02)** |  |  |  |  |  |  | 0.90 |
| M5 | **1.56*** (0.44)** | -0.03  (0.05) | 0.00 (0.00) |  | -0.00 (0.01) | **0.08*** (0.02)** | **0.05* (0.02)** | **0.30*** (0.05)** | 0.33 (0.27) | -0.25 (0.34) | 0.03 (0.30) | -0.02 (0.06) | 0.04 (0.08) | 0.05 (0.07) | 0.00 (0.00) | -0.00 (0.01) | -0.00 (0.01) |  |  |  | 0.90 |
| M6 | **1.81*** (0.45)** | **-0.33**  (0.11)** | **0.06** (0.02)** | **-0.01** (0.02)** | -0.00 (0.01) | **0.08*** (0.02)** | **0.05* (0.02)** | **0.30*** (0.05)** | 0.15 (0.29) | -0.44 (0.36) | 0.09 (0.32) | 0.20 (0.14) | 0.28 (0.18) | -0.01 (0.16) | -0.04 (0.03) | -0.05 (0.03) | 0.01 (0.03) | 0.00 (0.00) | 0.00 (0.00) | -0.00 (0.00) | 0.90 |
|  |  |  |  |  |  |  |  |  |  |  |  |  |  |  |  |  |  |  |  |  |  |
|  |  | Model Fit | | | | | |  |  |  |  |  |  |  |  |  |  |  |  |  |  |
|  |  | M1 | M2 | M3 | M4 | M5 | M6 |  |  |  |  |  |  |  |  |  |  |  |  |  |  |
|  | AIC | 5788.01 | 5799.91 | 5799.58 | 5814.09 | 5857.94 | 5891.10 |  |  |  |  |  |  |  |  |  |  |  |  |  |  |
|  | BIC | 5832.08 | 5849.49 | 5854.66 | 5891.21 | 5957.09 | 6012.29 |  |  |  |  |  |  |  |  |  |  |  |  |  |  |

**Note:** G1: 0 = control, 1 = social media abstinence, G2: 0 = control, 1 = gaming abstinence, G3: 0 = control, 1 = combined gaming and social media abstinence, AIC = Akaike Information Criterion, BIC = Bayesian Information Criterion, PSU = Problematic Smartphone use, FCV = Fear of COVID-19, BID = body image dissatisfaction, I = Intercept. **p* < .05, ***p* < .01, ****p* < .001.

**Supplementary Material 10** Daily measured FoMO

|  | Fixed effects | | | | | | | | | | | | | | | | | | | | | Random effects |
| --- | --- | --- | --- | --- | --- | --- | --- | --- | --- | --- | --- | --- | --- | --- | --- | --- | --- | --- | --- | --- | --- | --- |
|  | I | Day | Day² | Day³ | PSU | FCV | BID | Trait  FoMO | State  FoMO | G1 | G2 | G3 | Day * G1 | Day * G2 | Day * G3 | Day2 * G1 | Day² * G2 | Day² * G3 | Day³ * G1 | Day³ * G2 | Day³ * G3 | $\sigma_{u}^{2}$ |
| M1 | 0.20  (0.31) | **-0.01**  (0.00)** |  |  | 0.01  (0.01) | 0.03  (0.01) | 0.01  (0.01) | **0.25**  (0.08)** | 0.18  (0.11) |  |  |  |  |  |  |  |  |  |  |  |  | 0.44 |
| M2 | 0.25 (0.31) | **-0.04* (0.02)** | 0.00  (0.00) |  | 0.01  (0.01) | 0.03  (0.01) | 0.01  (0.01) | **0.25** (0.08)** | 0.18  (0.11) |  |  |  |  |  |  |  |  |  |  |  |  | 0.44 |
| M3 | 0.46  (0.32) | **-0.28*** (0.04)** | **0.05*** (0.01)** | **-0.01***  (0.01)** | 0.01  (0.01) | 0.03  (0.01) | 0.01  (0.01) | **0.24**  (0.08)** | 0.18  (0.11) |  |  |  |  |  |  |  |  |  |  |  |  | 0.44 |
| M4 | 0.07 (0.33) | **-0.02* (0.01)** |  |  | 0.01  (0.01) | 0.03  (0.01) | 0.02  (0.01) | **0.26** (0.08)** | 0.17  (0.11) | 0.14  (0.17) | -0.15  (0.22) | 0.23  (0.19) | 0.00  (0.01) | 0.02  (0.01) | 0.01  (0.01) |  |  |  |  |  |  | 0.43 |
| M5 | 0.07  (0.34) | -0.02  (0.03) | 0.00  (0.00) |  | 0.01  (0.01) | 0.03  (0.01) | 0.02  (0.01) | **0.26** (0.08)** | 0.17  (0.11) | 0.26  (0.19) | -0.22  (0.24) | 0.28  (0.21) | -0.05  (0.04) | 0.06  (0.06) | -0.02  (0.05) | 0.00  (0.00) | -0.00  (0.00) | 0.00  (0.00) |  |  |  | 0.43 |
| M6 | 0.20  (0.34) | **-0.16*  (0.08)** | **0.03* (0.01)** | **-0.01*  (0.01)** | 0.01  (0.01) | 0.03  (0.01) | 0.02  (0.01) | **0.26** (0.08)** | 0.17  (0.11) | 0.34  (0.20) | -0.08  (0.26) | **0.45* (0.23)** | -0.15  (0.10) | -0.11  (0.13) | -0.21  (0.11) | 0.02  (0.02) | 0.03  (0.02) | 0.04  (0.02) | -0.00  (0.00) | -0.00  (0.00) | -0.00  (0.00) | 0.43 |
|  |  |  |  |  |  |  |  |  |  |  |  |  |  |  |  |  |  |  |  |  |  |  |
|  | Model Fit | | | | | | |  |  |  |  |  |  |  |  |  |  |  |  |  |  |  |
|  |  | M1 | M2 | M3 | M4 | M5 | M6 |  |  |  |  |  |  |  |  |  |  |  |  |  |  |  |
|  | AIC | 4673.69 | 4685.33 | 4653.27 | 4705.92 | 4748.29 | 4754.55 |  |  |  |  |  |  |  |  |  |  |  |  |  |  |  |
|  | BIC | 4723.26 | 4740.41 | 4713.86 | 4788.54 | 4852.95 | 4881.24 |  |  |  |  |  |  |  |  |  |  |  |  |  |  |  |

**Note:** G1: 0 = control, 1 = social media abstinence, G2: 0 = control, 1 = gaming abstinence, G3: 0 = control, 1 = combined gaming and social media abstinence, AIC = Akaike Information Criterion, BIC = Bayesian Information Criterion, PSU = Problematic Smartphone use, FCV = Fear of COVID-19, BID = body image dissatisfaction, I = Intercept. **p* < .05, ***p* < .01, ****p* < .00
